# Enhancing circadian clock function in cancer cells inhibits tumor growth

**DOI:** 10.1186/s12915-017-0349-7

**Published:** 2017-02-14

**Authors:** Silke Kiessling, Lou Beaulieu-Laroche, Ian D. Blum, Dominic Landgraf, David K. Welsh, Kai-Florian Storch, Nathalie Labrecque, Nicolas Cermakian

**Affiliations:** 1 Douglas Mental Health University Institute, Montreal, QC H4H 1R3 Canada; 2 Department of Psychiatry, McGill University, Montreal, QC H3A 1A1 Canada; 3 Center for Circadian Biology and Department of Psychiatry, University of California, San Diego, CA 92037 USA; 4 Veterans Affairs San Diego Healthcare System, San Diego, CA 92161 USA; 5 Maisonneuve-Rosemont Hospital Research Centre, Montreal, QC H1T 2M4 Canada; 6 Department of Medicine, University of Montreal, Montreal, QC H3T 1J4 Canada; 7 Department of Microbiology, Infectiology and Immunology, University of Montreal, Montreal, QC H3T 1J4 Canada; 8 Present address: ZIEL Institute for Food and Health, Technical University of Munich, Freising, Germany

**Keywords:** Circadian clock, Clock gene expression, Tumor growth, Melanoma cancer, Cell cycle checkpoints

## Abstract

**Background:**

Circadian clocks control cell cycle factors, and circadian disruption promotes cancer. To address whether enhancing circadian rhythmicity in tumor cells affects cell cycle progression and reduces proliferation, we compared growth and cell cycle events of B16 melanoma cells and tumors with either a functional or dysfunctional clock.

**Results:**

We found that clock genes were suppressed in B16 cells and tumors, but treatments inducing circadian rhythmicity, such as dexamethasone, forskolin and heat shock, triggered rhythmic clock and cell cycle gene expression, which resulted in fewer cells in S phase and more in G1 phase. Accordingly, B16 proliferation in vitro and tumor growth in vivo was slowed down. Similar effects were observed in human colon carcinoma HCT-116 cells. Notably, the effects of dexamethasone were not due to an increase in apoptosis nor to an enhancement of immune cell recruitment to the tumor. Knocking down the essential clock gene *Bmal1* in B16 tumors prevented the effects of dexamethasone on tumor growth and cell cycle events.

**Conclusions:**

Here we demonstrated that the effects of dexamethasone on cell cycle and tumor growth are mediated by the tumor-intrinsic circadian clock. Thus, our work reveals that enhancing circadian clock function might represent a novel strategy to control cancer progression.

**Electronic supplementary material:**

The online version of this article (doi:10.1186/s12915-017-0349-7) contains supplementary material, which is available to authorized users.

## Background

Tumor cells are characterized by uncontrolled cell proliferation resulting in abnormal and accelerated tissue growth. In contrast, “healthy” cells often proliferate with a division rate of ~24 h [1]. This is due to the direct control of cell cycle checkpoints by the intracellular circadian clock machinery [2].

Circadian clocks operate in most tissues at the single-cell level [3]. At the molecular level, these clocks are based on clock genes, which participate in auto-regulatory feedback loops. In the core loop, the transcription factors CLOCK and BMAL1 activate the expression of *Per* and *Cry* genes, whose protein products negatively feed back on their own expression [4]. Several additional feedback loops contribute to this canonical mechanism, including one involving the nuclear receptor NR1D1. Moreover, in any given cell type, 5–20% of the transcriptome is under circadian control [5]. This is the basis for circadian control of major physiological processes, including immune functions and, most importantly for this investigation, cell proliferation [2, 6].

Misalignment between the external and internal time and circadian disruption, such as during shift work, has been associated with an increased cancer risk [7–10] and promotes tumor growth [11–13]. Moreover, circadian clock alteration due to mutations of single clock genes, such as *Per2* or *Bmal1*, accelerates tumor growth [14, 15] or the whole carcinogenesis process [16–18]. This is putatively due to an increase in proliferation rate upon circadian rhythm disruption, because tumor suppressor and key cell cycle genes are under clock control [2, 3] in the context of a bidirectional clock–cell cycle coupling [19, 20]. In support of the connection between rhythm disruption and oncogenesis, deregulated circadian rhythms appear to be a common feature of cancer cell lines [14, 21] and advanced-stage tumors [3]. Moreover, in mouse models of tumorigenesis, the outcome of the disease correlates with the level of improvement of circadian rhythmicity [22, 23]. Intriguingly, cancer prognosis and survival has been associated with the level of circadian disruption in patient tumor tissues [24, 25]. Although a previous study suggested a role for the circadian clock within cancer cells in tumor growth [22], direct evidence has been missing. Here, we show that improving circadian rhythms in the tumor slows down cell cycle progression and strongly reduces proliferation and tumor growth.

## Methods

### Study design

The general objective of the study was to investigate the role of the tumor cell-intrinsic circadian clock in relation to cell proliferation in culture and tumor growth in mice. For this purpose, we activated the circadian clock in B16 melanoma cells and subcutaneous (s.c.) B16 tumors and compared cell cycle gene expression, cell cycle phase distribution and tumor growth after repeated dexamethasone (DEX) or phosphate-buffered saline (PBS) treatment under controlled laboratory experimental conditions. We repeated those experiments using Control or *Bmal1* short hairpin RNA (shRNA)-transfected B16 tumors as a model with an inducible or non-inducible circadian clock. In the in vitro experiments, other clock-enhancing treatments (forskolin, heat shock) were also used. Further, we used NOD-*scid* IL2Rgamma^null^ (NSG) mice to exclude the possible role of DEX on immune infiltration in the tumors. HCT-116 cells and tumors were used to extend the data obtained from B16 melanoma cells to another cancer cell line, from human origin. In all animal experiments, mice were killed after 7–13 days of treatment and during the second day in constant darkness at the indicated circadian hours. The sample size could change during an experiment when the tumor size reached the previously defined clinical endpoint of individual mice and animals had to be killed. The sample size of all biological replicates per time point is indicated in each figure legend or the related tables (in Additional file 1), and mice were randomized between all groups. The study was not performed double-blinded: the experimenter was not blind to the identity of the animal in the different groups, because the treatment of each animal had to be performed according to the specific group. None of the animals was excluded from the analysis or the statistics.

### Cell culture and bioluminescence recordings

The B16 and HCT-116 cell lines, developed from murine skin and human colonic carcinoma [26, 27], were obtained from Drs Hua Gu (Institut de Recherche Clinique de Montréal, Montréal, QC, Canada) and Dindial Ramotar (University of Montréal, Montréal, QC, Canada), respectively, and cultured using standard conditions. Stable transfections with luciferase reporters were done according to standard procedures. More details can be found in the Additional file 2. All cell lines tested negative for *Mycoplasma*. B16 cells express the glucocorticoid receptor [28] and HSF1 [29], the latter being relevant for circadian synchronization by heat shock [30]. HCT-116 cells seem not to express functional glucocorticoid receptors [31]. However, steroids such as glucocorticoids can act through glucocorticoid receptor-independent pathways [32] and the synchronization of circadian rhythms in HCT-116 cells can be achieved by DEX treatment [33].

For whole-culture imaging, B16 cells or 200 μm slices of tumors from B16 cells with luciferase reporters were cultured in sealed dishes and recorded in a LumiCycle luminometer (Actimetrics Inc., Wilmette, IL, USA). Overall brightness was calculated by averaging values from 12 h after the start of recording to the end of recording. For single-cell imaging, B16 cells or B16 tumor slices were cultured under a microscope inside an incubator, and recorded using a charge-coupled device camera (Ikon-M 934BV Series, Andor Technology, Belfast, UK). Images were collected at intervals of 30 min, with 29 min exposure duration. Data were smoothed by a running minimum algorithm, and bioluminescence intensity was measured within a region of interest with a constant size, but defined manually for each moving cell. Detailed conditions and data analysis can be found in Additional file 2.

### ShRNA knockdown

*Bmal1* shRNA or Scrambled shRNA Lentiviral Particles (Creative Biogene, Shirley, NY, USA) consist of a pool of three constructs encoding 19–25 nt long target-specific shRNA, or shRNA with the same sequence composition, but scrambled. We ensured that the sequences of *Bmal1* shRNAs were absent in the mouse genome. B16 cells were grown in 12-well plates until 50% confluency. The medium was replaced with antibiotic-free Opti-MEM medium with 5 μg/mL Polybrene (Sigma-Aldrich, St. Louis, MO, USA). Cells were infected by the addition of 1 × 10^5^ infectious units of virus. After 24 h, the medium was replaced with regular growth medium. Stable clones expressing the shRNA were selected using puromycin (4 μg/mL) (Sigma-Aldrich). All cell lines tested negative for *Mycoplasma*.

### Proliferation, apoptosis and cell cycle assays in vitro

To study proliferation, cell death and cell cycle, B16 and HCT-116 cells were cultured until reaching 70% confluency and then stimulated with DEX (200 nM) or forskolin (FSK) (100 nM). Medium was replaced after 2 h. In different experiments, the cells were exposed to a heat shock of 43 °C for 30 min [30]. Live and dead cells were counted using Trypan blue, and apoptotic cells were assessed by Annexin V staining. Cell cycle phases were studied by bromodeoxyuridine (BrdU) and 7-aminoactinomycin D (7AAD) stainings, cell cycle phase distribution and cell cycle arrest were measured by Ki67 and Annexin V stainings, and mitotic index was assessed by phospho-histone H3 (pHH3) staining, using standard protocols. More details can be found in Additional file 2.

### Animals, DEX injections and tumor growth monitoring

C57BL/6J mice and NSG mice (Jackson Laboratories, Bar Harbor, ME, USA) were housed (≤5/cage) under 12 h light (100 lux):12 h dark (0 lux) conditions with food and water available ad libitum. Male mice (2–3 months old) were used in all experiments, except for one experiment with shRNA-expressing tumors to evaluate the tumor growth, in which 4-month-old females were used. The sample size of all biological replicates per time point is indicated in each figure legend or related supplementary table and mice were randomized between all groups.

C57BL/6J mice or NSG mice were inoculated with 1.5 × 10^6^ B16 cells in the tail vein or subcutaneously or 3 × 10^6^ HCT-116 cells. The s.c. tumor volume was measured in two dimensions with a caliper and calculated based on the volume of an ellipsoid. When s.c. tumors reached a volume of ~100 μL (after 10–15 days), DEX (or PBS) was injected intra-tumorally every second day at *Zeitgeber Time* (ZT) 6 (6 hours after lights on) to reach a concentration of 200 nM within the tumor (calculated based on the tumor volume). Tumor growth was measured daily. Tumor growth was compared showing absolute tumor volume when the tumors were on average 100 μL and did not differ between mice by more than approximately ±15 μL at the first treatment day. In case of experimental starting conditions when the tumor volume of mice differed more than 50 μL between individual mice at the first treatment day, the relative tumor growth was calculated relative to the initial starting volume for each individual mouse. Mice were killed by cervical dislocation at the indicated circadian times (CTs) during the second day of darkness, 18–25 days after inoculation. Eyes were removed under a 15 W red safety light prior to dissection. Tumors and host organs were harvested and kept at −80 °C until further analysis.

### Cell cycle, immune cell labeling and protein expression assays on tumors

Cell cycle phases were determined using BrdU and 7AAD staining. BrdU (1.2 mg/ml) was injected intra-tumorally 6 h before tumors were collected. Cell lysates from fixed s.c. tumors were incubated with mouse anti-BrdU antibody and then with Alexa Fluor 488 goat anti-mouse IgG. Finally, cells were incubated with 7AAD viability staining solution. Mitotic index was evaluated on tumor cell lysate incubated with anti-pHH3 antibody and then with Alexa Fluor 488 donkey anti-rabbit antibody. A second set of cell lysates were stained for Annexin V. Cell cycle arrest and proliferation was detected in cells from fixed s.c. tumors in NSG mice or in Scrambled shRNA and *Bmal1* shRNA cultured cells using the Ki67 Set according to manufacturer’s protocol.

For tumor immune cell infiltration in vivo, tumors were collected throughout the circadian day (second day in darkness) on the seventh day after the first DEX treatment. One million cells from s.c. tumor suspensions with and without collagenase treatment were blocked with Fc Block and then incubated with first antibodies for T cells (CD4, CD8), B cells (CD19), dendritic cells (CD11c), macrophages (F4/80, CD11b), neutrophils (CD11b, F4/80, Ly6G) or monocytes (CD11b, F4/80, Ly6C), followed by labeled streptavidin in the case of CD11b staining.

For protein expression in vivo, tumors were collected (as described above) and fixed in 4% paraformaldehyde, permeabilized with 90% methanol, and incubated with antibodies for WEE1, c-MYC, CYCLIN E (CCNE), CDK2, p57 (CDKN1c), CDK1, p21 (CDKN1a) or BMAL1 (ARNTL). After blocking with 10% rat or goat serum, appropriate secondary antibodies were used. Controls for each sample were stained with the secondary antibody alone. Finally, the cell suspensions were analyzed by flow cytometry.

All samples were analyzed by flow cytometry using a FACS Calibur (BD Biosciences, San Jose, CA, USA) and FlowJo software (FlowJo, LLC, Ashland, Oregon, USA). Controls for each sample were stained with the secondary antibody alone. More details can be found in Additional file 2, including panel descriptions, antibodies and fluorophores.

### Quantitative polymerase chain reaction and immunohistochemistry

Standard protocols were used to perform quantitative polymerase chain reaction (PCR) and immunohistochemistry. Details about protocols, primer sequences, antibodies and data analysis can be found in Additional file 2.

### Statistical analysis

Statistical analyses were done with GraphPad Prism (GraphPad Software, San Diego, CA, USA). Mann-Whitney rank sum test (alpha = 0.05, two-tailed) was used for knockdown efficiency. Circadian variation was tested by fitting a cosine-wave equation,

$$ \left[ y= Baseline+\left( Amplitude* \cos \left(2*\pi *\frac{x- Phaseshift}{24}\right)\right)\right], $$

on clock gene expression or a double harmonic cosine-wave equation,

$$ \left[ y= Baseline+\left( Amplitude1* \cos \left(2*\pi *\frac{x- Phaseshift1}{24}\right)\right)+\left( Amplitude2* \cos \left(4*\pi *\frac{x- Phaseshift2}{24}\right)\right)\right] $$

on cell cycle gene expression data, with a fixed 24-h period; significance was determined using an F-test. For datasets with more than two time points, a Kruskal-Wallis test was used (alpha = 0.05, two-tailed), taking the small sample size into consideration even if the normality test passed. For differences over time between two groups or more, two-way ANOVA was used, followed by Bonferroni’s posthoc test. A statistically significant difference was assumed when *p* < 0.05. Statistical details are presented in Additional file 1.

## Results

### Induction of circadian clock function in B16 melanoma cells

**Fig. 1:**
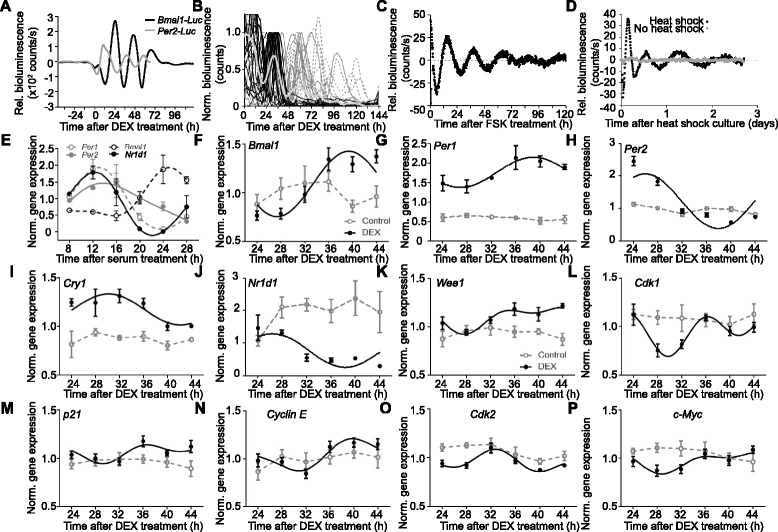
Induction of rhythmic clock gene and cell cycle gene expression in B16 cells. **a** Averaged *Bmal1-Luc* (*black line*) or *Per2-Luc* (*grey line*) bioluminescence after dexamethasone (*DEX*) treatment of B16 cells. **b** *Per2-Luc* bioluminescence of single B16 cells (*black*: one circadian peak, *grey*: two circadian peaks, *dashed grey*: more than two circadian peaks) and their average (*thick grey line*) after DEX treatment (n = 38). **c** Representative example of averaged *Per2-Luc* bioluminescence of B16 cells after forskolin (*FSK*) treatment. **d** Representative examples of averaged *Bmal1-Luc* bioluminescence of B16 cells after heat shock treatment (43 °C for 30 min, *black line*) and untreated controls (*grey line*). **e** Clock gene expression in B16 cells 8–28 h after serum shock (n = 19–23, 3–4 wells/time point; cosine-wave regression, F-test: *p* < 0.001). **f**–**j** Clock gene expression in cultured B16 cells 24–44 h after DEX treatment (*black line*). The control is shown in *grey*. Significant rhythms are illustrated with fitted cosine curves, otherwise data are connected by straight lines between data points, indicating no significant circadian rhythms (cosine-wave regression, F-test: control: all genes *p* > 0.05, DEX: *Per1* and *Cry1*: *p* < 0.05, n = 18, 3 wells/time point; *Per2* and *Bmal1: p* < 0.0001, n = 35–36, 5–6 wells/time point; *Nr1d1*: *p* < 0.001, n = 32–36, 5–6 wells/time point). **k**–**p** Expression of six cell cycle genes in B16 cells 24–44 h after DEX treatment. Significant rhythms are illustrated with fitted cosine curves, otherwise data are connected by straight lines between data points, indicating no significant circadian rhythms (n = 36, 6 wells/time point, multi-harmonic cosine-wave regression, F-test: control: *p* > 0.05, DEX: *p21* and *c-Myc*: *p* < 0.05; *Wee1* and *Cyclin E*: *p* < 0.01; *Cdk1* and *Cdk2*: *p* < 0.001). Data are represented as mean ± standard error of the mean. For details of statistics, see Additional file 1

First we characterized circadian rhythm generation in B16 melanoma cells. Reporter constructs consisting of the luciferase gene under the control of the *Bmal1* or the *Per2* promoter (*Bmal1*-*Luc*, *Per2*-*Luc*) were stably transfected in B16 cells. The bioluminescence of these cells was arrhythmic (Fig. 1a, from −44 to 0 h). However, upon addition of DEX, an agonist of the glucocorticoid receptor (which is expressed in B16 cells [28]) known to induce circadian rhythms in cultured cells, we observed rhythmic *Bmal1*-*Luc* and *Per2*-*Luc* promoter activity with the expected opposite phases (Fig. 1a) [34], which, however dampened earlier than in various non-cancer cell lines [35, 36].

To address whether the loss of rhythmicity after a few cycles resulted from circadian dysfunction in individual cells or from a desynchronization among rhythmic cells, we analyzed *Per2-Luc* bioluminescence at the single-cell level (Fig. 1b, Additional file 3, Additional file 4A–E). Although rhythms in the circadian range (first to second peak: 24.2 ± 1.4 h, second to third peak: 25.3 ± 2.0 h, third to fourth peak: 24.3 ± 1.5 h; Additional file 4E, F) were induced by DEX in ~90% of the cells, they quickly dampened within two to three cycles: 50% and 36% of the cells showed a second or third circadian peak, respectively, but circadian rhythms in bioluminescence were greatly suppressed in all cells after 132 h (Fig. 1b, Additional file 4A–D, G). Similarly the amplitude of single-cell rhythms dropped by about 80% within three cycles and the phase distribution became dispersed after 48 h (Additional file 4H, I). Repeated treatments showed that clock gene suppression was not due to cell death (Additional file 4J). Overall, these data indicated a suppression of clock gene expression in B16 cells rather than a desynchronization between single cells.



**Additional file 3:** Induction of rhythmic clock gene expression in B16 cells. *Per2-Luc* single-cell bioluminescence of cultured B16 cells for 142 h after dexamethasone treatment. (AVI 1831 kb)


Rhythmic clock gene expression was also observed when employing alternative methods for clock gene activation [30, 37]: FSK, an activator of adenylyl cyclase and of the cAMP/PKA pathway (Fig. 1c), heat shock (Fig. 1d) and serum shock (Fig. 1e) all induced transcript oscillations in B16 cells. In addition to *Bmal1* and *Per2*, the mRNAs of clock genes *Per1*, *Cry1* and *Nr1d1* also fluctuated rhythmically in B16 cells after DEX treatment but not in untreated cells (Fig. 1f–j).

Altogether, these results indicated that the B16 cells harbor an unstable but inducible circadian oscillator. We decided to take advantage of this property to address the role of the tumor cell-intrinsic clock in cell proliferation and tumor growth.

### The cell cycle is under circadian control after dexamethasone treatment in vitro

Because the molecular clockwork was shown to regulate the expression of genes encoding cell cycle regulators [2], we set out to study the expression of such genes in B16 cells. B16 cells were treated with DEX for 2 h, harvested over 24 h, and tested for mRNA expression of *WEE 1 homolog 1* (*Wee1*), *Cyclin-dependent kinase 1 (Cdk1), Cdk2*, *Cyclin E*, *Myelocytomatosis oncogene c* (*c-Myc*) and *Cyclin-dependent kinase inhibitor 1A* (*p21*)*.* All six genes showed predominant 24-h rhythm components in transcript abundance as a consequence of DEX treatment (Fig. 1k–p).

**Fig. 2:**
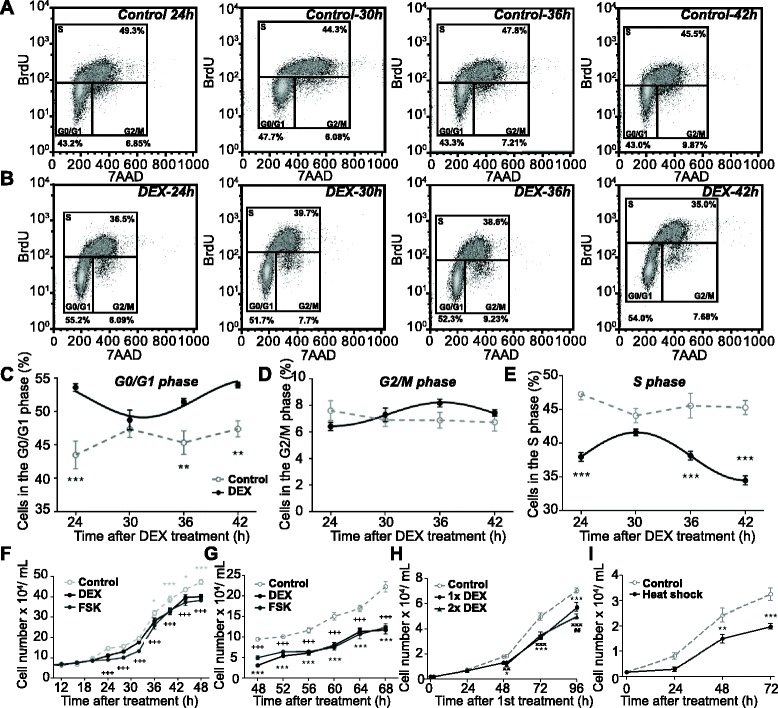
Dexamethasone (*DEX*), forskolin (*FSK*) and heat shock treatments reduce B16 cell proliferation. **a**, **b** Representative flow cytometry dot plots and **c**–**e** overall analysis for cell cycle phases in B16 cells 24–44 h after DEX treatment (or untreated), stained for incorporated BrdU and with 7AAD. Significant rhythms are illustrated with fitted cosine curves, otherwise data are connected by straight lines between data points, indicating no significant circadian rhythms (*n* = 24, 6 wells/time point, cosine-wave regression, F-test: Control: all genes/phases *p* > 0.05, DEX: *p* < 0.05; two-way ANOVA, posthoc test, group: **p < 0.01, ***p < 0.001). **f**, **g** Total alive cell numbers 12–48 h (**f**) and 48–68 h (**g**) after treatment with either DEX or FSK and controls (n = 6–8 wells/time point). **h** Alive cell numbers 0–96 h after control or DEX treatment at 0 h (1x DEX) or at 0 and 48 h (2x DEX) (n = 4–12 wells/time point). **i** Alive cell numbers 0–72 h after heat shock or control treatment (*n* = 6 wells/time point). Two-way ANOVA, posthoc test, *p < 0.05, **p < 0.01, ***p < 0.001 for DEX compared to controls; ^+++^ *p* < 0.001 FSK compared to controls; ^##^ *p* < 0.01 compared to 1x DEX; ^x^ *p* < 0.05, ^xx^ *p* < 0.01, ^xxx^ *p* < 0.001 compared to 2x DEX. Data are represented as mean ± standard error of the mean. For details of statistics, see Additional file 1

Because these factors are involved in cell cycle checkpoints, we assessed the effect of DEX treatment on the distribution of B16 cells among cell cycle stages. Cells were collected at different time points over 24 h, stained for BrdU incorporation and with 7AAD, and analyzed by flow cytometry (Fig. 2a, b). The proportion of cells in G0/1, G2/M and S phases was found to be rhythmic in cells treated with DEX, while control cells showed no circadian variation (Fig. 2c–e). Interestingly, fewer cells entered the S phase 24, 36 and 42 h after DEX treatment (Fig. 2e), indicating less DNA replication, while more cells were found in G0/G1 phases (Fig. 2c).

### Induction of the circadian clock slows down B16 cell proliferation in vitro

Given that activation of the circadian clock in B16 cells triggered rhythms of cell cycle genes and phases, we next tested whether activation of the clock in B16 cells influences their proliferation. Thirty-six hours after DEX treatment, we counted significantly fewer live cells than without treatment (Fig. 2f), while the amount of dead cells in the medium was unchanged after 12–48 h (Additional file 4N). The effect was even more pronounced after 2 days, with ~50% fewer live cells but similar numbers of dead cells after DEX treatment (Fig. 2g, Additional file 4O). Of note, the numbers of live and dead cells were similar before and immediately after the end of the 2 h DEX treatment, suggesting that the treatment did not acutely induce cellular toxicity (Additional file 4K).

Because rhythms quickly dampened after treatment (Fig. 1) we tested whether repeated DEX treatment could further inhibit cell proliferation. While a single DEX treatment significantly reduced cell numbers after 50 h, a second treatment further reduced the amount of cells after 96 h (Fig. 2h). Population doubling time (PDT) was increased to 22.7 h in single DEX-treated cells and further to 23.0 h in double DEX-treated cells compared to 16.5 h PDT in untreated control cells. Annexin V staining indicated that the differences in cell counts were not due to differences in levels of apoptosis (Additional file 4P). Total cell numbers were even more reduced after three DEX treatments administered every 48 h (Additional file 4L, M).

Similar experiments were also conducted with other stimuli activating the B16 clock (Fig. 1). Both a single FSK treatment (Fig. 2f, g) and repeated FSK treatments (Additional file 4M) were as effective as DEX in slowing down B16 cell proliferation. Also, FSK treatment did not affect cell death rates (Additional file 4N, O), nor did it acutely impact on cell numbers right after the 2 h treatment (Additional file 4K). Moreover, exposing the cells to a 30 min heat shock was sufficient to reduce proliferation without affecting apoptosis (Fig. 2i, Additional file 4Q). PDT was increased to 20.7 h after heat shock compared to 17.2 h in untreated cells. These data suggested that the reduction in cell proliferation might be due to action on intrinsic clock function, as activating clock gene expression was the common denominator of all three treatments.

### Dexamethasone activates the circadian clock in B16 tumors

**Fig. 3:**
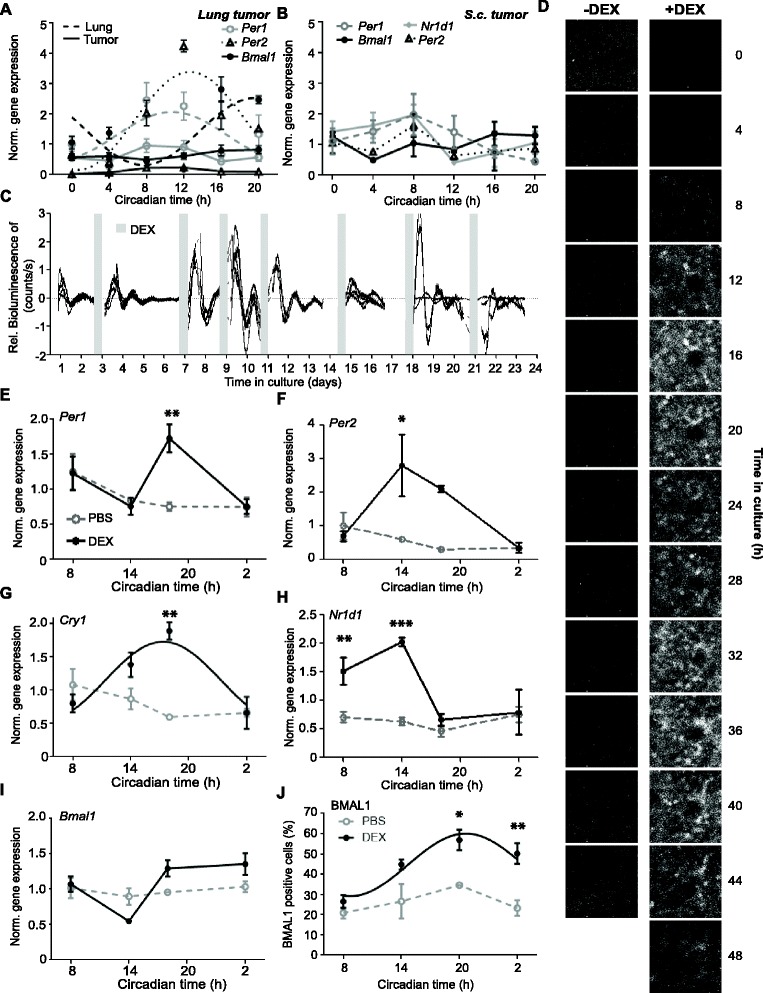
Dexamethasone (*DEX*) injection induces rhythmic clock gene expression in B16 tumors in vivo*.* **a**, **b** Clock gene expression in the lung and B16 lung tumors (a) (*n* = 21–22; cosine-wave regression: *Bmal1*: tumor: *p* > 0.05, lung: *p* < 0.01, n = 22, 3–4 mice/time point; *Per1*: tumor: *p* > 0.05, lung: *p* < 0.05; *Per2*: tumor: *p* < 0.05, lung: *p* < 0.0001; two-way ANOVA: interaction: *p* < 0.0001, group: *p* < 0.0001, Time: *p* < 0.0001) and subcutaneous (*s.c.*) tumors (b) (n = 15–16, 2–3 mice/time point, cosine-wave regression, F-test: all genes: *p* > 0.05). **c** Bioluminescence of a cultured *Bmal1*-*Luc* tumor slice treated repeatedly with DEX (*grey bars*). **d** Snapshots of *Bmal1-Luc* bioluminescence of single B16 cells in a lung tumor slice over 44 h in the absence of (*left panel*) or over 48 h after DEX treatment (*right panel*) from Additional file 6. **e**–**i** Clock gene expression in subcutaneous (s.c.) tumors after repeated intra-tumoral DEX or phosphate-buffered saline (PBS) injection (*n* = 9–10, 2–3 mice/time point; two-way ANOVA: time effect: *Per2* and *Cry1*: *p* < 0.05, *Per1* and *Nr1d1*: *p* < 0.01, *Bmal1*: *p* < 0.001; group effect: *Per1* and *Cry1*: *p* < 0.05, *Per2* and *Nr1d1*: *p* < 0.001, *Bmal1*: *p* < 0.05; posthoc test, group effect: *p < 0.0.5, **p < 0.01, ***p < 0.001). **j** BMAL1 protein expression in DEX- or PBS-treated tumors generated by s.c. injection (*n* = 11–12, 3 mice/time point, cosine-wave regression, F-test: PBS: *p* > 0.05, DEX: *p* < 0.01; two-way ANOVA: group *p* < 0.0001, time: *p* < 0.01) treated repeatedly for 8–11 days. Significant rhythms are illustrated with fitted cosine curves, otherwise data are connected by straight lines between data points, indicating no significant circadian rhythms. Data are represented as mean ± standard error of the mean. For details of statistics, see Additional file 1

B16 melanoma cells, which represent a well-established and widely used mouse model for human melanoma [38], form lung and s.c. tumors when injected in the tail vein and subcutaneously, respectively (Additional file 5A, B). The analysis of circadian clock gene expression in B16 lung tumors unveiled suppressed or arrhythmic *Per1*, *Per2* and *Bmal1* expression, while there was robust circadian oscillation in the neighboring lung tissue (Fig. 3a). Similarly, *Per1*, *Per2*, *Bmal1* and *Nr1d1* expression was arrhythmic in s.c. B16 tumors (Fig. 3b).

DEX, an agonist of the glucocorticoid receptor, is well known to reset cellular clocks by inducing *Per* gene expression [39]. Upon repeated addition of DEX, we observed rhythmic *Bmal1-Luciferase* reporter expression in slice cultures of explanted B16 lung tumors (Fig. 3c). Moreover, monitoring of single cells within the same lung tumor slice indicated an absence of *Bmal1* rhythms before treatment, but circadian oscillation of *Bmal1* after DEX treatment (Additional file 6, Fig. 3d). Thus, DEX induces de novo rhythmic *Bmal1* gene expression in single B16 cells rather than synchronizing cellular oscillators. This is consistent with the single-cell data obtained on B16 cells in vitro (Fig. 1, Additional files 3 and 4).



**Additional file 6:** Bioluminescence monitoring in the absence and after induction of rhythmic clock gene expression in cells of a B16 lung tumor. *Bmal1-Luc* single-cell bioluminescence in a B16 lung tumor slice for 44 h in the absence of DEX treatment and for 68 h after DEX treatment. (AVI 2935 kb)


To further confirm an activation of the tumor clock by DEX in vivo, s.c. tumors were injected intra-tumorally with DEX every 48 h to ensure maintenance of tumor clock activation. Rhythmic clock gene expression in tumors was found after DEX but not PBS treatment (Fig. 3e–i). *Per1*, *Per2*, *Cry1* and *Nr1d1* expression showed a significant effect of time and treatment. Rhythmicity of *Bmal1* mRNA expression did not reach significance (Fig. 3i). However, the ANOVA analysis showed an effect of time (see Additional file 1), and immunohistochemistry on s.c. tumor slices revealed a significant rhythm of BMAL1 protein levels in DEX-treated tumors, but not in PBS-treated tumors (Fig. 3j, Additional file 5C, D).

The efficiency and specificity of the intra-tumoral injections were evaluated by injecting methylene blue in tumors. The injected fluid spanned the whole tumor tissue 6 h after intra-tumoral injection, but was absent in surrounding tissues (Additional file 5E). To further assess the specificity of the response, clock gene expression in the liver - a non-cancerous, peripheral tissue - was compared between PBS- and DEX-injected mice: we found no significant differences between the treatment groups (Additional file 5F).

Altogether, these results indicated that the B16 tumors harbor a suppressed but inducible circadian oscillator. Moreover, these experiments showed that repeated DEX injection consistently induced rhythmic clock gene expression in B16 tumors rather than re-synchronized them. The ability to compare the B16 tumors with or without circadian clock function gave us the opportunity to test the role of the tumor-intrinsic clock in regulating cell cycle and tumor growth.

### The cell cycle is under circadian control after dexamethasone treatment of B16 tumors

**Fig. 4:**
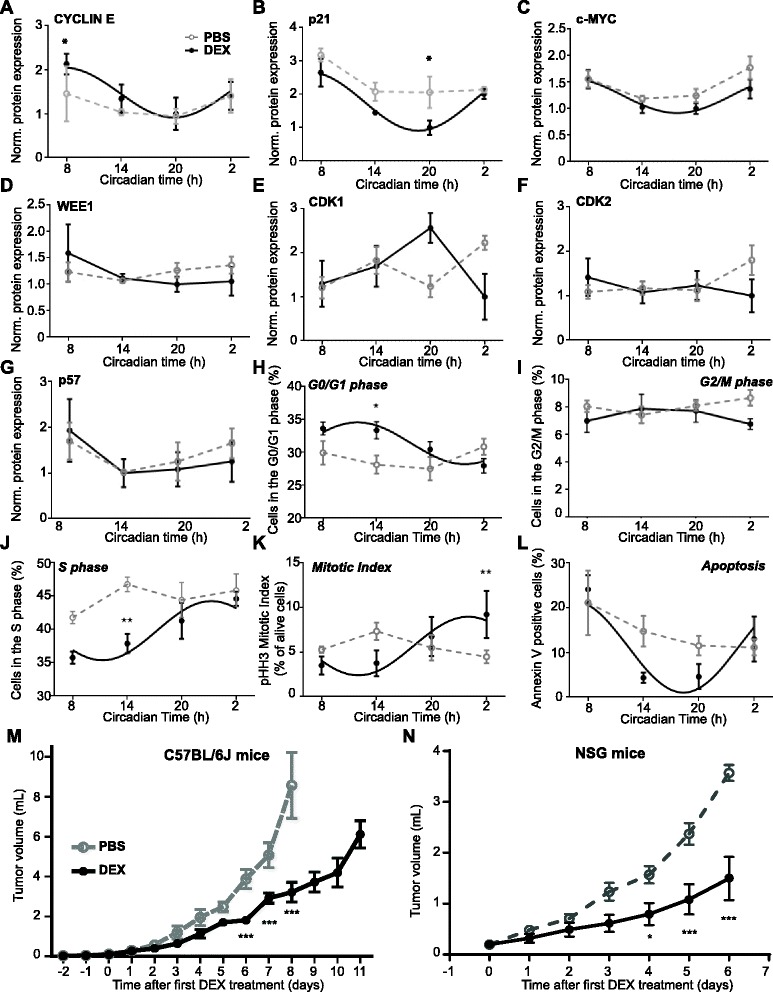
Dexamethasone (*DEX*) injection induces rhythmic cell cycle events and reduces B16 tumor growth in vivo*.* **a**–**g** Cell cycle protein expression in subcutaneous (s.c.) tumors after repeated intra-tumoral DEX or phosphate-buffered saline (*PBS*) injection every 2 days for 8–11 days (*n* = 16–18, 4–5 mice/time point; F-test: PBS: all genes: *p* > 0.05; DEX: CDK1, CDK2, p57: *p* > 0.05; c-MYC: *p* ≤ 0.05; WEE1, p21: *p* < 0.01; CYCLIN E: *p* < 0.001). **h**–**l** Analysis for cell cycle phase distribution and apoptosis in s.c. tumors (*n* = 15–18, 3–5 mice/time point; F-test: PBS all phases: *p* > 0.05, DEX: G2/M: *p* > 0.05; G0/G1, S, apoptosis: *p* < 0.05; mitotic index: *p* < 0.01). **m** Volume of s.c. tumors in C57BL/6 J mice injected intra-tumorally at day 0, 2, 4, 6, 8 and 10 with DEX or PBS (*n* = 3–11 mice/group/time point, two-way ANOVA: *p* < 0.0001; posthoc test: ***p < 0.001). Data are represented as mean ± standard error of the mean (SEM). **n** Volume of s.c. tumors in NSG mice injected intra-tumorally at day 0, 2, 4 and 6 with DEX or PBS (*n* = 4 mice/group, two-way ANOVA: *p* < 0.0001; posthoc test: *p < 0.05, ***p < 0.001). Data are represented as mean ± SEM. For details of statistics, see Additional file 1

We measured the expression of cell cycle regulators in s.c. B16 tumors harvested over 24 h. Similar to the data obtained in vitro (Fig. 1k–p), rhythmic protein expression of CYCLIN E, p21 and c-MYC was found in DEX-treated tumors, but not in PBS-treated tumors (Fig. 4a–c, Additional file 7A–C). On the other hand, WEE1, CDK1, CDK2 and p57, a direct glucocorticoid receptor target [40], did not show a daily variation in the DEX-treated tumors (Fig. 4d–g).

Homogenized tumor cells collected at different time points were stained for BrdU incorporation and with 7AAD. The proportion of cells in G0/1 and S phases was found to be rhythmic in DEX-treated control tumors, whereas PBS-treated tumors showed no circadian variation (Fig. 4h–j, Additional file 7D). Interestingly, fewer rhythmic cells entered the S phase in DEX-treated tumors, indicating less DNA replication, whereas more cells were found in G0/G1 phases. Importantly, DEX treatment reduced rather than increased the number of cells in G0 arrest as indicated by Ki67/7AAD staining in vitro (see control cells in Additional file 8). Consistent with results obtained from BrdU stainings, cell numbers in G1 phase were increased, while cells distributed in S/G2/M phases were slightly reduced (compare Figs. 2 and 4 and Additional file 8). Thus, reduced cell numbers after DEX treatment were not caused by cell cycle arrest. Moreover, even though entrance to G2/M phase was not rhythmically controlled in the tumor, mitotic index assessed by pHH3 staining indicated a rhythmic percentage of cells undergoing mitosis (Fig. 4k, Additional file 7E). Interestingly, cells from DEX-treated tumors underwent apoptosis in a circadian manner (Fig. 4l, Additional file 7F).

### Dexamethasone treatment slows down B16 tumor growth

Given that activation of the circadian clock in B16 tumors triggered rhythms of cell cycle genes and phases, we then evaluated whether the activation of clock function in B16 tumors was paralleled by a reduction in tumor growth. To this end, s.c. B16 tumor growth was compared between DEX- and PBS-treated tumors. DEX treatment significantly slowed down tumor growth (by ~60% after 8 days) compared to PBS treatment (Fig. 4m). Importantly though, Annexin V staining indicated that the differences in tumor growth were not due to differences in the levels of apoptosis, because a similar proportion of apoptotic cells were found in DEX- and PBS-treated tumors (when averaged over the 24-h day, PBS: 14.6 ± 2.3% versus DEX: 11.5 ± 4.6, extra sum-of squares F-test: *p* = 0.25; Fig. 4l).

To rule out that reduced tumor growth after DEX treatment may be caused by DEX-induced immune cell infiltration in the tumor, we repeated this experiment in immune-deficient NSG mice, which lack T cells, B cells and natural killer cells. Similarly to results in C57BL/6J hosts, tumor growth was strongly reduced by DEX in NSG mice (Fig. 4n), and no differences were found in the levels of infiltration of remaining immune cells between DEX- and PBS-treated tumors in NSG mice (Additional file 9A–C). Again, the proportion of cells undergoing apoptosis was not significantly different between DEX- and PBS-treated tumors collected at Circadian Time (CT)14 in NSG mice (Additional file 9D). Moreover, the proportions of cells undergoing cell division (G1/S/G2/M) and of those in G0 arrest were unchanged by DEX treatment of tumors in NSG mice (Additional file 9E). Together with results obtained in vitro that excluded cell cycle arrest (Additional file 8A) and in C57BL/6J mice that showed more cells in G0/G1 phase at CT14 upon DEX treatment, these results indicated that cells remain more in G1 phase and transit less to the S phase.

### Knockdown of *Bmal1* abolishes the effects of dexamethasone on clock genes and cell cycle genes

**Fig. 5:**
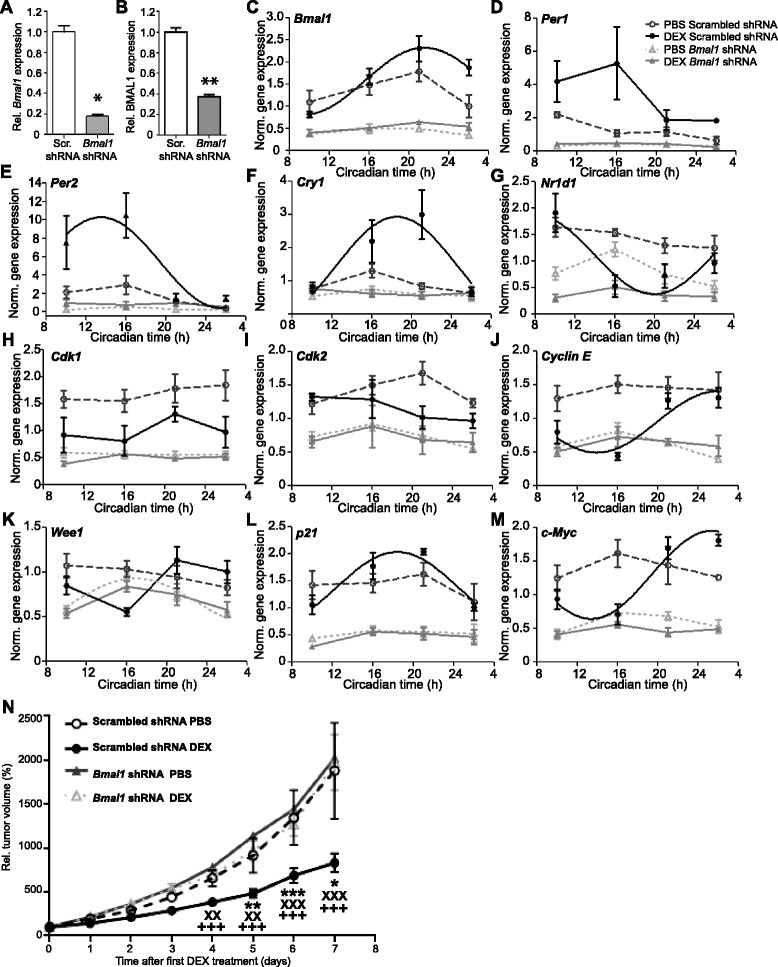
*Bmal1* knockdown prevents dexamethasone (*DEX*)-induced circadian rhythms and effects on tumor growth in vivo. **a**, **b** *Bmal1* knockdown efficiency was evaluated for gene expression by real-time PCR (*n* = 3–7, Mann-Whitney rank sum test: *p* < 0.05) (a) and flow cytometry for protein expression (*n* = 6, Mann-Whitney rank sum test: *p* < 0.01) (b). **c**–**g** Clock gene expression in DEX- or phosphate-buffered saline (PBS)-treated tumors generated by subcutaneous (s.c.) injection of Scrambled shRNA- or *Bmal1* shRNA-transfected B16 cells treated every 2 days for 11–13 days. Significant rhythms are illustrated with fitted cosine curves, otherwise data are connected by straight lines between data points, indicating no significant circadian rhythms. (n = 15–16, 3–4 mice/time point; two-way ANOVA: *Bmal1*, *Per2* and *Cry1*: group: *p* < 0.0001, time: *p* < 0.0001; *Per1*: group: *p* < 0.0001, time: *p* > 0.05; *Nr1d1*: group: *p* < 0.0001, time: *p* < 0.001). **h**–**m** Cell cycle gene expression in DEX- or PBS-treated tumors generated by s.c. injection of Scrambled shRNA- or *Bmal1* shRNA-transfected B16 cells. Significant rhythms are illustrated with fitted cosine curves, otherwise data are connected by straight lines between data points, indicating no significant circadian rhythms (*n* = 14–16, 3–4 mice/time point; two-way ANOVA: group: all genes: *p* < 0.0001, time: *Cdk1* and *Cyclin E*: *p* > 0.05, *Cdk2* and *Wee1*: *p* < 0.05, *p21* and *c-Myc*: *p* < 0.01). **n** Relative tumor volume of Scrambled shRNA or *Bmal1* shRNA s.c. tumors injected intra-tumorally with DEX or PBS at day 0, 2, 4 and 6. Scrambled shRNA tumors: *n* = 7–22, two-way ANOVA, *p* < 0.0001; *Bmal1* shRNA tumors: *n* = 10–20, two-way ANOVA: *p* > 0.05 (no significant difference between DEX and PBS throughout the experiment). Data are represented as mean ± standard error of the mean. For details of statistics, see Additional file 1

To test whether the activation of the clock in B16 tumors is the causal link between DEX treatment and the inhibition of tumor growth, experiments were repeated in B16 tumors with a disrupted circadian clock. Using a lentiviral vector, we stably introduced into B16 cells an shRNA against *Bmal1*, a necessary component of the circadian clock [4]. *Bmal1* shRNA-transfected cells expressed ~80% less *Bmal1* RNA and ~65% less BMAL1 protein than Scrambled shRNA control cells (Fig. 5a, b). B16 cells stably expressing shRNA against *Bmal1* or Scrambled shRNA were injected subcutaneously into mice to form either tumors lacking a functional clock or control tumors. Similarly to non-transfected tumors, clock gene expression showed significant circadian rhythms in DEX-treated but not PBS-treated Scrambled shRNA tumors (compare Fig. 3 and Fig. 5c–g). In *Bmal1* shRNA tumors*, Bmal1* expression was reduced by ~70% in both DEX- and PBS-treated tumors (Fig. 5c). Accordingly, the expression of BMAL1 target genes *Per1*, *Per2*, *Cry1* and *Nr1d1* was suppressed and arrhythmic, indicating effective disruption of the clock machinery in the tumors (Fig. 5d–g). Thus, *Bmal1* knockdown completely prevented the induction of circadian rhythms by DEX in the *Bmal1* shRNA tumors.

Rhythmic gene expression of *Cyclin E*, *p21* and *c-Myc* was only found in DEX-treated Scrambled shRNA tumors; *Cdk1*, *Cdk2* and *Wee1* did not show a daily variation, in line with cell cycle protein data (compare Fig. 5h–m and Fig. 4a–f). Importantly, abrogation of DEX-induced activation of the circadian clock in *Bmal1* shRNA tumors was reflected by the arrhythmic expression of cell cycle genes in these tumors. Consistently, *Bmal1* knockdown in vitro also prevented the DEX-induced rhythms in cell cycle phases (Additional file 8A–E).

### Knockdown of *Bmal1* prevents the inhibitory effect of dexamethasone on tumor growth

Next we sought to determine whether clock function in the B16 tumors is needed for DEX-induced reduction in tumor growth. As expected, B16 tumors expressing the Scrambled shRNA grew more slowly during DEX treatment: after 7 days, DEX-treated tumors were ~60% smaller than PBS-treated tumors (Fig. 5n) and this is consistent with the growth of untransfected tumors (Fig. 4m, n). In contrast, tumor volumes were indistinguishable between DEX- and PBS-treated mice harboring *Bmal1* shRNA tumors (Fig. 5n). Thus, DEX had no effect on tumor growth after *Bmal1* knockdown. Volume doubling time analysis of Scrambled and *Bmal1* shRNA tumors confirmed these results (Scrambled shRNA: PBS: 53.3 h, DEX: 84.0 h; *Bmal1* shRNA: PBS: 50.1 h, DEX: 50.0 h). Similar results were obtained in vitro using *Bmal1* and Scrambled shRNA-transfected cells (data not shown).

Consistent with the data obtained using NSG mice (Fig. 4n), DEX did not affect the levels of immune cell infiltration in B16 tumors inoculated in the syngeneic C57BL/6J mice or in *Bmal1* knockdown tumors, indicated by similar values for B cells, dendritic cells, CD4+ and CD8+ T cells, macrophages, neutrophils, and monocytes (Additional file 8F–L). These data confirmed that the slower tumor growth was not caused by effects of DEX on immune cell infiltration. Overall, these findings are in strong support of the notion that activating circadian clock function within B16 tumors slows down their growth.

### Enhancement of circadian rhythms slows down HCT-116 tumor growth

**Fig. 6:**
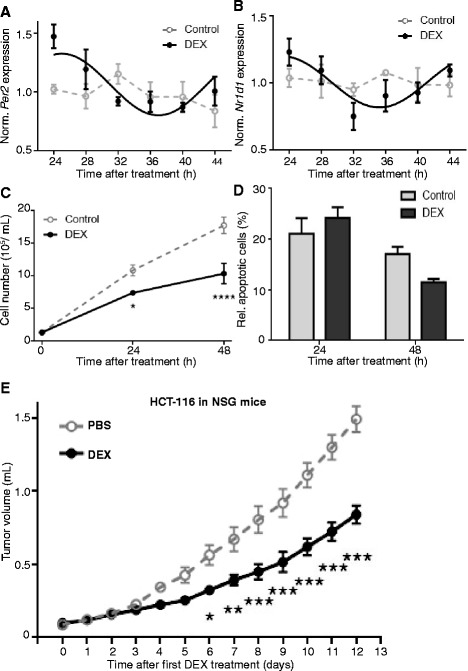
Dexamethasone (*DEX*) induces circadian rhythms and reduces HCT-116 cell proliferation and tumor growth in vivo. **a**, **b** *Per2* and *Nr1d1* gene expression in cultured HCT-116 cells 24–44 h after DEX treatment. Significant rhythms are illustrated with fitted cosine curves, otherwise data are connected by straight lines between data points, indicating no significant circadian rhythms (*n* = 17–18, 3–4 wells/time point, cosine-wave regression, F-test: DEX: *p* < 0.05, control: *p* > 0.05). **c**, **d** Cell numbers (c) and apoptotic cells stained with Annexin V (d) 24–72 h after DEX or control treatment (*n* = 5–6 wells/time point). Two-way ANOVA, posthoc test, *p < 0.05, ****p < 0.0001. **e** Volume of HCT-116 subcutaneous tumors in NSG mice injected intra-tumorally at day 0, 2, 4, 6, 8, 10 and 12 with DEX or phosphate-buffered saline (*PBS*) (*n* = 5 mice/group/time point, two-way ANOVA, *p* < 0.0001). Data are represented as mean ± standard error of the mean. For details of statistics, see Additional file 1

To test the link between the circadian clock and tumor growth beyond our model using mouse melanoma, we treated human HCT-116 colon carcinoma cells with DEX and measured clock gene expression as well as proliferation and apoptosis up to 48 h after the treatment. HCT-116 cells exhibited rhythmic clock gene expression upon DEX treatment (Fig. 6a, b), which could be due to either synchronization of individual cells’ clocks or de novo activation of clocks in the cells. Upon this treatment HCT-116 cell proliferation was strongly decreased while apoptosis levels were unaffected (Fig. 6c, d).

Similar to the B16 s.c. tumors in C57BL/6 J and NSG mice, growth of tumors formed in NSG mice after HCT-116 cell inoculation was strongly slowed down after DEX treatment (Fig. 6e). These data underscore the possibility that controlling cell division rate by circadian clock enhancement and thus controlling tumor growth is not restricted to mouse melanoma cells, and may be generally applicable to human cancer cells and to other cancer types.

## Discussion

**Fig. 7:**
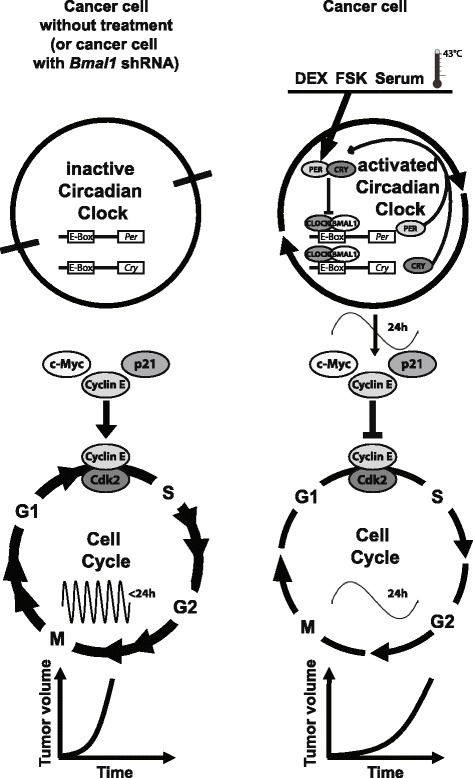
Model for the suppressive effect of clock activation on tumor growth. Untreated cancer cells harbor an inactive circadian clock and exhibit fast cell cycle progression and fast tumor growth (*left*). Dexamethasone (*DEX*), Forskolin (*FSK*), serum shock or heat shock activate the circadian clock in cancer cells, which regulates circadian expression of cell cycle checkpoint genes (in particular factors regulating G1-to-S transition), which results in slower tumor growth (*right*). Treatment of cancer cells transfected with *Bmal1* shRNA does not result in an activation of the circadian clock and thus no rhythmic cell cycle gene expression, which results in fast tumor growth (*left*). See Discussion for details

In this report, we showed that B16 melanoma cells and B16 tumors have suppressed expression of clock components, which is in line with previous reports on different cancer cell lines and tumors [21–23]. However, we discovered that different treatments could restore clock gene expression in B16 cells and tumors. This provided an experimental model in which the same tumor type could be compared in conditions of inactive or active circadian oscillator function. Using this model, we showed that restoring clock function in B16 melanoma cells strongly reduced cell proliferation in vitro and tumor growth in vivo (Fig. 7). We also extended our observations to another cancer cell model, the human colon carcinoma HCT-116 cells. In all of the in vivo tumor experiments, a strong slowdown of tumor growth was observed, with a two thirds reduction of tumor size after 8 days of treatment, regardless of the host or cancer cell line used.

The amplitude of clock gene rhythmicity within tumors was previously correlated with their growth in mice bearing Glasgow osteosarcoma [22] and pancreatic adenocarcinoma [15]. Furthermore, a dysregulated circadian tumor clock in human patients correlated with their prognosis [25, 41]. Together, these prior studies indicate that the tumor clock may have an impact on tumor growth and overall survival (also reviewed in [42]). However, until our present report, a causal link was missing to solidify the importance of the tumor clock itself, as opposed to host-derived circadian rhythms. Previous studies showed that glucocorticoids suppress proliferation in human melanoma cells and B16 tumors after intraperitoneal DEX injection in mice [28, 43]. Importantly, our data provide a cellular mechanism for these effects. In contrast to prior work, our data were generated using intra-tumor injection of DEX restricted to the tumor and at low doses. Furthermore, we showed an absence of effects on clock gene expression in the liver. Thus, our work is the first to show a tumor-intrinsic mechanism for the control of tumor growth by the circadian clock. Moreover, the effects of restored synchronous circadian rhythmicity on tumor growth also extend to a distinct tumor cell model, the human colon carcinoma HCT-116 cells, although in the case of these cells we cannot exclude a role of DEX in synchronizing the clocks in individual cells rather than an activation of the cellular clocks.

DEX and FSK have previously been reported to have varying effects on apoptosis, depending on the cell line or tumor model used [44–47]. Nevertheless, and in line with previous reports on fibroblasts [44], we found no or minor effects of DEX, FSK or heat shock on apoptosis in B16 cells. Thus, in our experiments, the activation of circadian clock function in tumors resulted in reduced growth independently of deregulated apoptosis. Given the immune-modulating properties of glucocorticoids [48], we also aimed to exclude an effect of DEX on the immune cell content of the tumors as the cause of the observed decrease in tumor growth. Notably, immune cell content was not changed by the intra-tumoral DEX treatment. Moreover, the effect of DEX on tumor growth was recapitulated in NSG mice, which lack T cells, B cells and natural killer cells, further strengthening our claim. In order to directly test the hypothesis that DEX inhibits tumor growth via the tumor-intrinsic circadian clock, we used shRNA against the essential clock gene *Bmal1*. The response to DEX was abrogated in B16 tumors with a *Bmal1* knockdown. Thus, the suppressive DEX effect on B16 proliferation and tumor growth requires a functional clock within B16 cells. It is unlikely that *Bmal1* knockdown affects the response to DEX itself, because *Bmal1* dysfunction did not interfere with glucocorticoid receptor expression as observed in transcriptome datasets [49–51]. Of note, we observed a similar induction of circadian rhythms and inhibition of B16 cell proliferation in vitro following other treatments besides DEX, as well as with a non-pharmacological treatment, heat shock. Serum, DEX, FSK and heat shock all reset cellular clocks by inducing clock genes such as *Per* genes [30, 39, 52], but different signaling pathways are involved: DEX activates *Per1* via the glucocorticoid receptor [53], but DEX can also synchronize the circadian clock via glucocorticoid receptor-independent pathways, as was shown for HCT-116 cells [33]; FSK activates the cAMP/protein kinase A pathway, and thus, cAMP response element-binding protein activity at the *Per1* promoter [54]; and heat shock activates *Per2* transcription via heat shock factor 1 [30]. Given that these treatments similarly activate the clock by mechanisms distinct from DEX, this further suggests a clock-dependent action of DEX.

Taken together our results indicate that reduced tumor growth after DEX treatment was not caused by enhanced apoptosis, nor immune infiltration in the tumor, but rather relies specifically on inducing circadian rhythms within the tumor. For the first time, our work unveils a causal relationship between the tumor-intrinsic circadian clock and tumor growth (Fig. 7).

Our findings raise the question of how the activation of circadian clock function in B16 cells leads to inhibition of tumor growth. The circadian clock has been shown to regulate cell cycle genes and, thus, progression through the cell cycle [2]. Indeed, we identified rhythmic cell cycle genes after induction of clock function in B16 cells and tumors. In particular, *Cyclin E*, *p21* and *c-Myc* exhibited circadian oscillations at the mRNA and protein levels. CLOCK-BMAL1 represses *c-Myc*, an activator of *Cyclin E* [16, 55], and another clock component, NR1D1, inhibits the synthesis of the CDK inhibitor *p21* [56]. Thus, restored expression of the major clock components in B16 tumors likely accounts for the observed changes in cell cycle gene expression.

Our data indicate that the circadian gating distributes more B16 cells in the G1 phase (and not more arrest of cells in the G0 phase) and fewer in the S phase, which prolongs the transit through the cell cycle and may account for the observed lengthening of the PDT. Thus, fewer cells initiated DNA replication, which could underlie the observed reduced tumor growth. In line with our observations, accumulation in cell cycle phases such as G1 or G2 was reported to slow down proliferation in human melanoma cells [43]. G1-to-S transit is regulated by cell cycle checkpoint regulators, in particular the Cyclin E/CDK2 complex [57]. Importantly we have identified rhythmic expression of the inhibitor p21, c-MYC and a component of this checkpoint complex itself, Cyclin E. Consequently, rhythmic regulation of the Cyclin E/Cdk2 complex may result in a prolonged stay in the G1 phase. Taken together, rhythmic changes in expression levels of Cyclin E, p21 and c-MYC after activation of the clock by DEX likely caused the observed redistribution of B16 cells among cell cycle phases (Fig. 7). Moreover, our results highlight a possible strategy of B16 melanoma cells to facilitate their proliferation: suppression of the circadian clock induces changes in their cell cycle, which leads to increased residence time in the S phase and an enhancement of DNA replication and thus cell proliferation.

## Conclusions

Glucocorticoids have been used to treat various cancer types for decades, in many cases as an adjuvant to other chemotherapies [58]. While potential mechanisms have been described in different cell types, exactly how glucocorticoids regulate apoptosis or cell cycle progression is not fully understood [59, 60]. Our work indicates a possible mechanism (capable of explaining existing data) for how glucocorticoids can inhibit cancer cell proliferation, namely by inducing circadian rhythms in cancer cell cycle events to limit the number of cells in the S phase and thereby increase the time spent in the G1 phase. Therefore, our work opens new windows of opportunity in cancer therapy based on chronobiological intervention. For example, activation of the clock in tumors may represent a new therapeutic target for malignant melanoma therapy in humans. Because our observations extended to human colon cancer cells, this strategy might more generally become an innovative way to slow down cancer progression and thereby improve the outcome of established anti-cancer therapies. Slowing down tumor progression by improving circadian rhythmicity might critically delay cancer progression and/or metastasis. This could in turn increase treatment success with traditional chemotherapy or provide a broader time window for surgical resection of tumors.

## Additional files

Additional file 1: Supplementary tables. (PDF 362 kb)
Additional file 2: Supplementary methods. (PDF 184 kb)
Additional file 4: Single-cell analysis of *Per2-Luc* B16 cells and level of apoptosis after treatment. Bioluminescence of single *Per2-Luc* B16 cells after DEX treatment, grouped depending on their number of circadian peaks: (**A**) one peak (*n* = 19), (**B**) two peaks (*n* = 10), (**C**) three or more peaks (*n* = 9) and (**D**) abnormal and non-circadian peaks (n = 6). (**E**) Period distribution of single *Per2-Luc* B16 cells after DEX treatment. Analysis of circadian period (**F**), rhythmicity (**G**)*,* amplitude (**H**) and peak time (**I**) of single *Per2-Luc* B16 cells. (**J**) Bioluminescence of *Per2-Luc* B16 cells undergoing repeated treatments (grey bars) after plating: medium change (day 1), DEX (day 4, 7) and FSK (day 10). (**K**) B16 cells were counted before, 2 h after treatment with DEX or FSK (untreated: *n* = 24, DEX: n = 6, FSK: *n* = 6, Kruskal-Wallis test, *p* > 0.05), (**L**) after a single or three DEX treatments and compared to untreated controls (*n* = 6 wells/time point, two-way ANOVA *p* < 0.001, posthoc test, **p* < 0.05, ****p* < 0.001 DEX1 compared to controls, ^+++^ *p* < 0.001 DEX3 compared to controls, ^x^ *p* < 0.05 DEX1 compared to DEX3). (**M**) B16 cells were counted after a single or three DEX or FSK treatments and compared to untreated cells (control *n* = 25, DEX1: *n* = 16, DEX3: *n* = 4, FSK3: *n* = 4, Kruskal-Wallis test, Dunn’s multiple comparison test, ***p* < 0.01, ****p* < 0.001). (**N**, **O**) Total dead cells in the medium 12–48 h (N) and 48–68 h (O) after treatment with either DEX or FSK and controls (*n* = 6–8 wells/time point). (**P**) Apoptotic cells stained for Annexin V 0–96 h after control or DEX treatment at 0 h (DEX1) or at 0 and 48 h (DEX2) (*n* = 4–12 wells/time point). (**Q**) Apoptotic cells stained for Annexin V 0–72 h after heat shock or control treatment (*n* = 6 wells/time point). Two-way ANOVA, posthoc test, ****p* < 0.001 DEX compared to controls, ^+++^ *p* < 0.001 FSK compared to controls. For details of statistics, see Additional file 1. (PDF 1581 kb)
Additional file 5: B16 tumors and clock gene expression. (**A**) Lung tumors (black, indicated by arrows) formed after injection of 1.5 × 10^6^ B16 cells in the tail vein, within the surrounding lung tissue (pink). (**B**) B16 tumors (black, indicated by arrow) formed after subcutaneous injection of 1.5 × 10^6^ B16 cells in the neck. (**C**, **D**) Representative immunohistochemistry images for BMAL1 (green) and DAPI (blue) from PBS-treated (C) and DEX-treated (D) tumors collected at CT2, 8, 14 and 20. See full data in Fig. 3j. (**E**) Methylene blue (200 μL of 1% in saline) was injected intra-tumorally. Six hours later, tumors were harvested, sectioned and visualized by light microscopy. (**F**) Relative circadian clock gene expression in the liver of DEX- or PBS-treated mice harboring tumors generated by s.c. injection of B16 cells (*n* = 9–10, 2–3 mice/time point; cosine-wave regression, F-test: -DEX: *Bmal1* and *Nr1d1*: *p* < 0.05, +DEX: *Bmal1* and *Cry1*: *p* ≤ 0.01, *Per1*: *p* = 0.348, *Per2*: *p* = 0.174, *Nr1d1*: *p* = 0.0646; two-way ANOVA: group: all genes: *p* > 0.05, time: *Bmal1*, *Per2*, *Cry1* and *Nr1d1*: *p* < 0.001; *Per1*: *p <* 0.01). Significant rhythms are illustrated with fitted cosine curves, otherwise data are connected by straight lines between data points, indicating no significant circadian rhythms. Data are represented as mean ± SEM. For details of statistics, see Additional file 1. (PDF 8 MB)
Additional file 7: DEX induces rhythmic cell cycle protein expression and cell cycle phases, mitosis and apoptosis. Representative flow cytometry dot plots for p21 (**A**), c-MYC (**B**) and CYCLIN E (**C**) protein expression at the indicated CTs in s.c. tumors after repeated intra-tumoral DEX or PBS injection every 2 days for 8–11 days. Unstained control (red) and stained (blue) cells of the indicated antibody. (**D**) Representative flow cytometry dot plots for incorporated BrdU and staining with 7AAD at the indicated CTs in s.c. tumors after repeated intra-tumoral DEX or PBS injection every 2 days for 8–11 days. The illustrated gates indicate the analyzed cell cycle phases. Representative histogram for pHH3 (**E**) and histogram for Annexin V staining (**F**) at the indicated CTs in s.c. tumors after repeated intra-tumoral DEX or PBS injection every 2 days for 8–11 days. Positive (red) cells compared to unstained control cells (black). (PDF 2 MB)
Additional file 8: Knockdown of *Bmal1* prevents the DEX effects on B16 cell proliferation, cell cycle arrest and cell cycle phases. (**A**, **B**) Cell cycle arrest of Scrambled shRNA-transfected B16 cells (A) or *Bmal1* shRNA-transfected B16 cells (B) indicated by cells in the G0 phase (left panels) and cell proliferation indicated by cells in the G1 phase (middle panels) or S/M/G2 phases (right panels) with control or DEX treatment (*n* = 5–6, wells/time point/group; two-way ANOVA, posthoc test, group effect: **p* < 0.0.5, ***p* < 0.01, ****p* < 0.001). (**C**–**E**) Cell cycle phases of Scrambled shRNA- and *Bmal1* shRNA-transfected B16 cells after DEX treatment. Significant rhythms are illustrated with fitted cosine curves, otherwise data are connected by straight lines between data points, indicating no significant circadian rhythms (*n* = 10–15, 3–4 wells/time point; cosine-wave regression, F-test: *p* < 0.05; two-way ANOVA, posthoc test, group effect: ***p* < 0.01, ****p* < 0.001). Frequencies of (**F**) B cells (CD19^+^), (**G**) dendritic cells (CD11c^+^), (**H**) CD4 T cells (CD4^+^), (**I**) CD8 T cells (CD8^+^), (**J**) macrophages (F4/80^high^Ly6C^-^, gated on CD11b^+^), (**K**) neutrophils (*F4*/*80* ^low^Ly6G^high^, gated on CD11b^+^) and (**L**) monocytes (CD11b^high^Ly6C^+^, gated on F4/80^+^) relative to alive cells after intra-tumoral PBS or DEX injection in B16 s.c. tumors of C57BL/6 J mice (Scrambled shRNA tumors and *Bmal1* shRNA tumors: no significant difference between DEX and PBS throughout the experiment, *n* = 5–6 mice/group, Mann-Whitney rank sum test: *p* > 0.05). Data are represented as mean ± SEM. For details of statistics, see Additional file 1. (PDF 844 kb)
Additional file 9: Immune cell infiltration, apoptosis and cell cycle arrest in NSG mice. Representative flow cytometry dot plots and quantification for neutrophils (*F4*/*80* ^low^Ly-6G^high^, gated on CD11b^+^) (**A**), monocytes (CD11b^high^Ly-6C^high^, gated on F4/80^+^) (**B**), macrophages (F4/80^high^Ly-6C^-^ gated on CD11b^+^) (**C**), histogram and quantification for apoptosis (Annexin V) stained (red) and unstained (grey) cells (**D**) and dot plots and quantification of proliferation (Ki67) (**E**) at the indicated CTs in s.c. tumors after repeated intra-tumoral PBS or DEX injection every 2 days for 8–11 days in B16 s.c. tumors of NSG mice collected at CT14 (*n* = 4 mice/group, Mann-Whitney rank sum test: *p* > 0.05). (PDF 812 kb)
