# Supplementary material for: Enhancing circadian clock function in cancer cells inhibits tumor growth
Source: BMC Biol. 2017 Feb 14;15:13. doi: 10.1186/s12915-017-0349-7 (PMC5310078; doi:10.1186/s12915-017-0349-7)
Supplement: Additional file 1: — Supplementary tables. (PDF 362 kb) [file 12915_2017_349_MOESM1_ESM.pdf]

Additional File 1: Tables

Detailed statistics for Figure 1. Induction of rhythmic clock gene and cell cycle gene expression in B16 cells

Panel E: Clock gene expression in B16 cells 8-28 h after serum shock.

1. **Cosine wave regression**

Gene	p-value (F-test)	Sample size
Per1	0.0004	19
Per2	0.0008	19
Bmal1	0.0002	20
Nr1d1	0.0006	23

Panels F-J: Clock gene expression in cultured B16 cells 24-44 h after DEX treatment.

1. **Cosine wave regression**

	p-value (F-test)		
**Gene**	**-DEX**	**+DEX**	**Sample size**
Bmal1	0.362	<0.0001	35 (-DEX), 36 (+DEX)
Per1	0.561	0.012	18, 18
Per2	0.723	<0.0001	36 , 36
Nr1d1	0.584	0.001	32, 36

2. **2-way ANOVA**

	p-value (2-way ANOVA)		
**Gene**	**Interaction**	**Group**	**Time**
Bmal1	< 0.0001	0.0159	< 0.0001
Per1	0.0056	< 0.0001	0.0725
Per2	< 0.0001	< 0.0001	< 0.0001
Nr1d1	0.0047	< 0.0001	0.4353

Panels K-P: Expression of six cell cycle genes in B16 cells 24-44 h after dexamethasone (DEX) treatment.

1. **Harmonic cosine wave regression**

	p-value (F-test)		
**Gene**	**-DEX**	**+DEX**	**Sample size**

Wee1	0.696	0.012	36
Cdk1	0.969	0.001	36
p21	0.847	0.045	36
Cyclin E	0.745	0.004	36
Cdk2	0.126	0.0009	36
c-Myc	0.633	0.050	36

2. **2-way ANOVA**

	**p-value (2-way ANOVA)**		
**Gene**	**Interaction**	**Group**	**Time**
Wee1	0.0188	< 0.0001	0.2304
Cdk1	0.1374	0.0049	0.0875
p21	0.1602	< 0.0001	0.5166
Cyclin E	0.4367	0.1142	0.0034
Cdk2	0.3319	< 0.0001	0.0007
c-Myc	0.0169	0.0013	0.9463

Detailed statistics for Figure 2. Dexamethasone, forskolin and heat shock treatments reduce B16 cell proliferation.

Panels C-E: Analysis for cell cycle phases

1. **Cosine wave regression**

	**p-value (F-test)**		
**Phase**	**-DEX**	**+DEX**	**Sample size**
G0/G1	0.905	0.002	24 (-DEX), 24 (+DEX)
G2/M	0.875	0.018	24 (-DEX), 24 (+DEX)
S	0.739	<0.0001	24 (-DEX), 24 (+DEX)

2. **2-way ANOVA**

	**p-value (2-way ANOVA)**		
**Phase**	**Interaction**	**Group**	**Time**
G0/G1	0.0161	<0.0001	0.1807
G2/M	0.1038	0.4036	0.7332
S	0.0009	<0.0001	0.0191

Panel F: Total alive cell numbers 12-48 h after treatment with either dexamethasone (DEX) or forskolin (FSK) and controls.

p-value (2-way ANOVA)					
**Interaction**	**Group**	**Time**			
<0.0001	<0.0001	<0.0001			
**p-value (Bonferroni posttest)**			**Sample Size**		
**Time (h)**	**Control vs DEX**	**Control vs FSK**	**Control**	**DEX**	**FSK**
12	>0.05	>0.05	6	6	6
16	>0.05	>0.05	6	6	6
20	>0.05	>0.05	6	6	6
24	>0.05	<0.001	6	6	6
28	>0.05	<0.001	6	6	6
32	>0.05	<0.001	6	6	6
36	<0.05	<0.001	6	6	6
40	<0.001	<0.001	6	6	6
44	<0.05	<0.001	6	6	6
48	<0.001	<0.001	6	6	6

Panel G: Total alive cell numbers and 48-68 h after treatment with either dexamethasone (DEX) or forskolin (FSK) and controls.

p-value (2-way ANOVA)					
**Interaction**	**Group**	**Time**			
<0.0001	<0.0001	<0.0001			
**p-value (Bonferroni posttest)**			**Sample Size**		
**Time (h)**	**Control vs DEX**	**Control vs FSK**	**Control**	**DEX**	**FSK**
48	<0.001	<0.001	8	8	8
52	<0.001	<0.01	8	8	8
56	<0.001	<0.001	8	8	8
60	<0.001	<0.001	8	8	8
64	<0.001	<0.001	8	8	8
68	<0.001	<0.001	8	8	8

Panel H: Alive cell numbers 0-96 h after DEX or control treatment at 0 h (DEX 1) or at 0 and 48 h (DEX 2)

p-value (2-way ANOVA)						
Interaction	Group	Time				
<0.0001	<0.0001	<0.0001				
p-value (Bonferroni posttest)				Sample Size		
Time (h)	Control vs DEX 1	Control vs DEX 2	DEX 1 vs DEX 2	Control	DEX 1	DEX 2
0	>0.05	>0.05	>0.05	12	12	12
2	>0.05	>0.05	>0.05	6	6	6
24	>0.05	>0.05	>0.05	6	6	6
48	>0.05	>0.05	>0.05	6	6	6
50	<0.05	<0.01	>0.05	6	6	6
72	<0.001	<0.001	>0.05	6	6	6
96	<0.001	<0.001	<0.01	6	4	6

Panel I: Alive cell numbers 0-72 h after heat shock or control treatment.

p-value (2-way ANOVA)			
Interaction	Group	Time	
0.0225	<0.0001	<0.0001	
p-value (Bonferroni posttest)		Sample Size	
Time (h)	Control vs Heat shock	Control	Heat shock
0	>0.05	3	3
2	>0.05	6	6
24	<0.01	6	6
96	<0.001	6	6

Detailed statistics for Figure 3. Dexamethasone injection induces rhythmic clock gene expression in B16 tumors *in vivo.*

Panel A: Clock gene expression in the lung and B16 lung tumors.

1. **Cosine wave regression / 2-way ANOVA**

	p-value (F-test)		
Gene	Lung	Tumor	Sample size
Bmal1	0.002	0.232	22 (lung), 22 (tumor)
Per1	0.031	0.187	21, 22

Per2	<0.0001	0.031	22, 21

p-value (2-way ANOVA)		
**Interaction**	**Group**	**Time**
< 0.0001	< 0.0001	< 0.0001

Panel B: Clock gene expression in the s.c. tumor tissue.

2. **Cosine wave regression**

	p-value (F-test)	
**Gene**	**Tumor**	**Sample size**
Bmal1	0.556	15
Per1	0.079	16
Per2	0.920	15
Nr1d1	0.084	16

3. **2-way ANOVA**

p-value (2-way ANOVA)		
**Interaction**	**Gene**	**Time**
0.7144	0.3200	0.0076

Panel E-I: Clock gene expression in s.c. tumors after repeated intra-tumoral dexamethasone (DEX) or PBS injection.

1. **Cosine wave regression**

	p-value (F-test)		
**Gene**	**PBS**	**DEX**	**Sample size**
Bmal1	0.877	0.096	9 (PBS), 10 (DEX)
Per1	0.256	0.940	9 (PBS), 10 (DEX)
Per2	0.319	0.093	10
Cry1	0.331	0.028	10
Nr1d1	0.474	0.097	10

2. **2-way ANOVA**

	p-value (2-way ANOVA)		
**Gene**	**Interaction**	**Group**	**Time**
Bmal1	0.0135	0.1626	0.0008
Per1	0.0109	0.0189	0.0039
Per2	0.0105	0.0006	0.0357
Cry1	0.0062	0.0122	0.0429
Nr1d1	0.0029	0.0002	0.0017

Panel J: BMAL1 protein expression in DEX- or PBS-treated tumors generated by s.c. injection.

4. **Cosine wave regression / 2-way ANOVA**

	p-value (F-test)		
**Protein**	**PBS**	**DEX**	**Sample size**
BMAL1	0.485	0.010	11 (PBS), 12 (DEX)

	p-value (2-way ANOVA)		
**Protein**	**Interaction**	**Group**	**Time**
BMAL1	0.1703	<0.0001	0.0038

Detailed statistics for Figure 4. DEX induces rhythmic cell cycle events and reduces B16 tumor growth *in vivo.*

Panels A-G: Cell cycle protein expression in s.c. tumors after repeated intra-tumoral DEX or PBS injection every 2 days for 8-11.

1. **Cosine wave regression**

	p-value (F-test)		
	Scrambled shRNA		
**Protein**	**PBS**	**DEX**	**Sample size**
WEE1	0.540	0.110	16 (PBS), 18 (DEX)
c-MYC	0.062	0.050	16, 18
p21	0.178	0.003	16, 18
CDK1	0.868	0.25	16, 18
CDK2	0.417	0.966	16, 16
CYCLIN E	0.190	0.0009	16, 18
p57	0.0543	0.123	16, 18

2-way ANOVA

	p-value (2-way ANOVA)		
**Protein**	**Interaction**	**Group**	**Time**
WEE1	0.1118	0.6875	0.1556
c-MYC	0.6083	0.0654	0.0030
p21	0.4504	0.0074	0.0002
CDK1	0.0253	0.9589	0.3790
CDK2	0.0264	0.4288	< 0.0001
CYCLIN E	0.1880	0.0375	0.0003
p57	0.4924	0.5435	0.0030

Panels H-L: Overall analysis for cell cycle phase distribution in DEX- or control- (PBS) treated tumors generated by s.c. injection of scrambled shRNA- or *Bmal1* shRNA-transfected B16 cells.

1. **Cosine wave regression**

	**p-value (F-test)**		
	**Scrambled shRNA**		
**Phase or measure**	**PBS**	**DEX**	**Sample size**
G0/G1	0.530	0.020	15 (PBS), 17 (DEX)
G2/M	0.445	0.743	15, 17
S	0.789	0.029	15, 17
pHH3	0.193	0.006	15, 17
Apoptosis	0.426	0.024	15, 18

2. **2-way ANOVA**

	**p-value (2-way ANOVA)**		
**Phase or measure**	**Interaction**	**Group**	**Time**
G0/G1	0.0475	0.0303	0.1697
G2/M	0.4353	0.1653	0.9570
S	0.1782	0.0006	0.0095
pHH3	0.0009	0.8007	0.0641
Apoptosis	0.2325	0.2575	0.0053

Panels M: Volume of B16 s.c. tumors in C57BL/6 mice injected intratumorally with DEX or PBS.

2-way ANOVA for C57BL/6 mice

**p-value (2-way ANOVA)**			
**Interaction**	**Group**	**Time**	
< 0.0001	< 0.0001	< 0.0001	
**p-value (Bonferroni posttest)**		**Sample Size**	
**Time (h)**	**PBS vs DEX**	**PBS**	**DEX**
-2	>0.05	7	6
-1	>0.05	5	6
0	>0.05	10	9
1	>0.05	8	8
2	>0.05	9	11

3	>0.05	10	7
4	>0.05	7	11
5	>0.05	7	9
6	<0.0001	6	7
7	<0.0001	6	7
8	<0.0001	5	3

Panels N: Volume of B16 s.c. tumors in NSG mice injected intratumorally with DEX or PBS.

2-way ANOVA for NSG mice

**p-value (2-way ANOVA)**			
**Interaction**	**Group**	**Time**	
< 0.0001	0.0129	< 0.0001	
**p-value (Bonferroni posttest)**		**Sample Size**	
**Time (h)**	**PBS vs DEX**	**PBS**	**DEX**
0	>0.05	4	4
1	>0.05	4	4
2	>0.05	4	4
3	>0.05	4	4
4	<0.05	4	4
5	<0.001	4	4
6	<0.0001	4	4

Detailed statistics for Figure 5. *Bmal1* knockdown prevents dexamethasone-induced circadian rhythms and effects on tumor growth *in vivo.*

Panels C-G: Clock gene expression in dexamethasone (DEX)- or PBS-treated tumors generated by s.c. injection of Scrambled shRNA- or *Bmal1* shRNA-transfected B16 cells treated every 2 days for 11-13 day.

1. **Cosine wave regression**

	**p-value (F-test)**				
	**Scrambled shRNA**		***Bmal1* shRNA**		
**Gene**	**PBS**	**DEX**	**PBS**	**DEX**	**Sample size**
Bmal1	0.236	0.001	0.196	0.156	13-15
Per1	0.061	0.380	0.370	0.414	13-16
Per2	0.110	0.042	0.377	0.957	13-16
Cry1	0.064	0.029	0.591	0.704	13-16
Nr1d1	0.294	0.041	0.037	0.661	13-16

2. 2-way ANOVA

	p-value (2-way ANOVA)		
**Gene**	**Interaction**	**Group**	**Time**
Bmal1	0.0003	<0.0001	<0.0001
Per1	0.281	<0.0001	0.079
Per2	0.0001	<0.0001	<0.0001
Cry1	<0.0001	<0.0001	<0.0001
Nr1d1	<0.0001	<0.0001	0.001

Bonferroni's posthoc test

		p-value (Bonferroni's posthoc test)			
		**Scrambled shRNA**	***Bmal1* shRNA**	**Scrambled vs *Bmal1* shRNA**	
**Gene**	**CT**	**PBS vs. DEX**	**PBS vs. DEX**	**PBS vs. PBS**	**DEX vs DEX**
Bmal1	10	> 0.05	> 0.05	<0.01	> 0.05
	16	> 0.05	> 0.05	<0.001	<0.001
	22	> 0.05	> 0.05	<0.001	<0.001
	26	<0.01	> 0.05	<0.05	<0.001
Per1	10	> 0.05	> 0.05	> 0.05	<0.01
	16	<0.001	> 0.05	> 0.05	<0.001
	22	> 0.05	> 0.05	> 0.05	> 0.05
	26	> 0.05	> 0.05	> 0.05	> 0.05
Per2	10	<0.01	> 0.05	> 0.05	<0.001
	16	<0.001	> 0.05	> 0.05	<0.001
	22	> 0.05	> 0.05	> 0.05	> 0.05
	26	> 0.05	> 0.05	> 0.05	> 0.05
Cry1	10	> 0.05	> 0.05	> 0.05	> 0.05
	16	> 0.05	> 0.05	> 0.05	<0.01
	22	<0.001	> 0.05	> 0.05	<0.001
	26	> 0.05	> 0.05	> 0.05	> 0.05
Nr1d1	10	> 0.05	> 0.05	<0.01	<0.001
	16	<0.001	> 0.05	> 0.05	> 0.05
	22	> 0.05	> 0.05	> 0.05	> 0.05
	26	> 0.05	> 0.05	> 0.05	> 0.05

Panels H-M: Cell cycle gene expression in dexamethasone (DEX)- or PBS-treated tumors generated by s.c. injection of Scrambled shRNA- or *Bmal1* shRNA-transfected B16 cell.

1. **Cosine wave regression**

	p-value (F-test)				
	Scrambled shRNA		*Bmal1* shRNA		
**Gene**	**PBS**	**DEX**	**PBS**	**DEX**	**Sample size**
Wee1	0.571	0.141	0.0007	0.090	13-16
c-Myc	0.676	0.003	0.153	0.754	13-16
p21	0.788	0.007	0.818	0.2447	13-16
Cdk1	0.768	0.885	0.986	0.368	13-16
Cdk2	0.176	0.474	0.045	0.873	13-16
Cyclin E	0.895	0.008	0.009	0.605	13-16

2. **2-way ANOVA**

	p-value (2-way ANOVA)		
**Gene**	**Interaction**	**Group**	**Time**
Wee1	<0.0001	<0.0001	0.042
c-Myc	<0.0001	<0.0001	0.003
p21	0.092	<0.0001	0.001
Cdk1	0.994	<0.0001	0.743
Cdk2	0.796	<0.0001	0.047
Cyclin E	<0.0001	<0.0001	0.169

3. **Bonferroni's posthoc test**

		p-value (Bonferroni's posthoc test)			
		Scrambled shRNA	*Bmal1* shRNA	Scrambled vs *Bmal1* shRNA	
**Gene**	**CT**	**PBS vs. DEX**	**PBS vs. DEX**	**PBS vs. PBS**	**DEX vs DEX**
Wee1	10	> 0.05	> 0.05	<0.001	> 0.05
	16	<0.01	> 0.05	> 0.05	> 0.05
	22	> 0.05	> 0.05	> 0.05	> 0.05
	26	> 0.05	> 0.05	> 0.05	<0.05
c-Myc	10	> 0.05	> 0.05	<0.001	> 0.05
	16	<0.001	> 0.05	<0.001	> 0.05
	22	> 0.05	> 0.05	<0.01	<0.001
	26	> 0.05	> 0.05	<0.001	<0.01
p21	10	> 0.05	> 0.05	<0.01	<0.001
	16	> 0.05	> 0.05	<0.001	<0.001
	22	> 0.05	> 0.05	> 0.05	> 0.05
	26	> 0.05	> 0.05	> 0.05	> 0.05

Cdk1	10	> 0.05	> 0.05	<0.01	<0.05
	16	<0.05	> 0.05	<0.01	> 0.05
	22	> 0.05	> 0.05	<0.001	<0.05
	26	<0.05	> 0.05	<0.001	> 0.05
Cdk2	10	> 0.05	> 0.05	> 0.05	<0.05
	16	> 0.05	> 0.05	<0.05	> 0.05
	22	<0.05	> 0.05	<0.001	> 0.05
	26	> 0.05	> 0.05	<0.05	> 0.05
Cyclin E	10	<0.05	> 0.05	<0.001	> 0.05
	16	<0.001	> 0.05	<0.01	> 0.05
	22	> 0.05	> 0.05	<0.001	<0.05
	26	> 0.05	> 0.05	<0.001	<0.05

Panel N: *Bmal1* knockdown prevents the effect of dexamethasone on tumor growth.

Normalized volume of Scrambled shRNA or *Bmal1* shRNA tumors relative to starting volume injected intra-tumorally with DEX or PBS. Results include data from two independent experiments.

**p-value (2-way ANOVA)**		
**Interaction**	**Group**	**Time**
<0.0001	<0.0001	<0.0001

**p-value (Bonferroni posttest)**						
**Time (days)**	**PBS vs DEX Srambled shRNA**	**PBS *Bmal1* shRNA vs DEX *Bmal1* shRNA**	**PBS Srambled vs PBS *Bmal1* shRNA**	**PBS Srambled vs DEX *Bmal1* shRNA**	**DEX Srambled vs PBS *Bmal1* shRNA**	**DEX Srambled vs DEX *Bmal1* shRNA**
1	>0.05	>0.05	>0.05	>0.05	>0.05	>0.05
2	>0.05	>0.05	>0.05	>0.05	>0.05	>0.05
3	>0.05	>0.05	>0.05	>0.05	>0.05	>0.05
4	>0.05	>0.05	>0.05	>0.05	>0.05	>0.05
5	>0.05	>0.05	>0.05	>0.05	<0.01	<0.001
6	<0.01	>0.05	>0.05	>0.05	<0.01	<0.001
7	<0.001	>0.05	>0.05	>0.05	<0.001	<0.001

**Sample size**				
**Time (days)**	**PBS Srambled shRNA**	**DEX Srambled shRNA**	**PBS *Bmal1* shRNA**	**DEX *Bmal1* shRNA**
1	22	22	20	19
2	22	22	20	19

3	22	21	20	18
4	22	20	20	18
5	17	20	20	18
6	13	18	19	16
7	10	13	18	13
1	7	13	12	10

Detailed statistics for Figure 6. Dexamethasone induces circadian rhythms and reduces HCT-116 cell proliferation and tumor growth *in vivo*.

Panels A, B: *Per2* and *Nr1d1* gene expression in cultured HCT-116 cells 24-44 h after dexamethasone (DEX) treatment.

1. **Cosine wave regression**

	**p-value (F-test)**		
**Gene**	**PBS**	**DEX**	**Sample size**
Per2	0.520	0.013	18
Nr1d1	0.999	0.033	18

2. **2-way ANOVA for *Per2***

**p-value (2-way ANOVA)**			
**Interaction**	**Group**	**Time**	
0.0444	0.1968	0.0325	
**p-value (Bonferroni posttest)**		**Sample Size**	
**Time (h)**	**PBS vs DEX**	**PBS**	**DEX**
24	<0.05	3	3
28	>0.05	3	3
32	>0.05	3	3
36	>0.05	3	3
4o	>0.05	3	3
44	>0.05	3	3

3. 2-way ANOVA for *Nr1d1*

p-value (2-way ANOVA)			
Interaction	Group	Time	
0.2183	0.8751	0.1018	
p-value (Bonferroni posttest)		Sample Size	
Time (h)	PBS vs DEX	PBS	DEX
24	>0.05	3	3
28	>0.05	3	3
32	>0.05	3	3
36	>0.05	3	3
4o	>0.05	3	3
44	>0.05	3	3

Panel C: Cell numbers 24-72 h after DEX or control (-DEX) treatment.

p-value (2-way ANOVA)			
Interaction	Group	Time	
0.0014	< 0.0001	<0.0001	
p-value (Bonferroni posttest)		Sample Size	
Time (h)	-DEX vs +DEX	-DEX	+DEX
0	>0.05	3	3
24	<0.05	6	6
48	<0.0001	6	6

Panel D: Apoptotic cells stained for Annexin V 24-72 h after DEX or control (-DEX) treatment.

p-value (2-way ANOVA)			
Interaction	Group	Time	
0.0556	0.5609	0.0010	
p-value (Bonferroni posttest)		Sample Size	
Time (h)	-DEX vs +DEX	-DEX	+DEX
24	>0.05	6	6
48	>0.05	6	6

Panel E: Volume of HCT-116 s.c. tumors in NSG mice injected intratumorally with DEX or PBS.

2-way ANOVA for NSG mice

**p-value (2-way ANOVA)**			
**Interaction**	**Group**	**Time**	
< 0.0001	0.0032	< 0.0001	
**p-value (Bonferroni posttest)**		**Sample Size**	
**Time (h)**	**PBS vs DEX**	**PBS**	**DEX**
0	>0.05	5	5
1	>0.05	5	5
2	>0.05	5	5
3	>0.05	5	5
4	>0.05	5	5
5	>0.05	5	5
6	<0.05	5	5
7	<0.01	5	5
8	<0.001	5	5
9	<0.0001	5	5
10	<0.0001	5	5
11	<0.0001	5	5
12	<0.0001	5	5
13	<0.0001	5	5

Detailed statistics for Additional File 4, Single cell analysis of bioluminescence of *Per2-Luc* B16 cells after dexamethasone treatment.

Panel L: B16 cells were counted after a single or 3 DEX treatments and compared to untreated controls

**p-value (2-way ANOVA)**						
**Interaction**	**Group**	**Time**				
0.0006	< 0.0001	<0.0001				
**p-value (Bonferroni posttest)**				**Sample Size**		
**Time (h)**	**DEX 1 vs -DEX**	**DEX 3 vs -DEX**	**DEX 1 vs DEX 3**	**Control**	**DEX 1**	**DEX 3**
96	>0.05	<0.001	>0.05	6	6	6
120	<0.05	<0.0001	>0.05	6	6	6
144	<0.001	<0.0001	<0.05	6	6	6

Panel M: B16 cells were counted after a single or 3 DEX or FSK treatments and compared to untreated cells

p-value (Kruskal-Wallis test)		<0.0001				
p-value (Dunn's Multiple Comparison Test)			Sample Size			
-DEX vs DEX 1	-DEX vs DEX 3	-DEX vs FSK 3	Control	DEX 1	DEX 3	FSK 3
<0.001	<0.001	<0.01	25	16	4	4

Panel N: Total dead cells in the medium 12-48 h after treatment with either dexamethasone (DEX) or forskolin (FSK) and controls.

p-value (2-way ANOVA)					
Interaction	Group	Time			
<0.0001	0.1599	<0.0001			
p-value (Bonferroni posttest)			Sample Size		
Time (h)	Control vs DEX	Control vs FSK	Control	DEX	FSK
12	>0.05	>0.05	6	6	6
16	>0.05	>0.05	6	6	6
20	>0.05	>0.05	6	6	6
24	>0.05	>0.05	6	6	6
28	>0.05	>0.05	6	6	6
32	>0.05	>0.05	6	6	6
36	>0.05	>0.05	6	6	6
40	>0.05	<0.001	6	6	6
44	<0.001	>0.05	6	6	6
48	>0.05	<0.001	6	6	6

Panel O: Total dead cells in the medium 48-68 h after treatment with either dexamethasone (DEX) or forskolin (FSK) and controls.

p-value (2-way ANOVA)					
Interaction	Group	Time			
0.0799	0.6562	0.8432			
p-value (Bonferroni posttest)			Sample Size		
Time (h)	Control vs DEX	Control vs FSK	Control	DEX	FSK
48	n/a	n/a	8	8	8
52	n/a	n/a	8	8	8
56	n/a	n/a	8	8	8
60	n/a	n/a	8	8	8
64	n/a	n/a	8	8	8
68	n/a	n/a	8	8	8

n/a, not applicable

Panel P: Apoptotic cells stained for Annexin V 0-96 h after DEX or control treatment at 0 h (DEX 1) or at 0 and 48 h (DEX 2)

p-value (2-way ANOVA)						
Interaction	Group	Time				
0.9573	0.5047	<0.0001				
p-value (Bonferroni posttest)				Sample Size		
Time (h)	Control vs DEX 1	Control vs DEX 2	DEX 1 vs DEX 2	Control	DEX 1	DEX 2
0	n/a	n/a	n/a	12	12	12
2	n/a	n/a	n/a	6	6	6
24	n/a	n/a	n/a	6	6	6
48	n/a	n/a	n/a	6	6	6
50	n/a	n/a	n/a	6	6	6
72	n/a	n/a	n/a	6	6	6
96	n/a	n/a	n/a	6	3	6

n/a, not applicable

Panel Q: A apoptotic cells stained for Annexin V 0-72 h after heat shock or control treatment.

p-value (2-way ANOVA)			
**Interaction**	**Group**	**Time**	
0.0495	0.4285	<0.0001	
**p-value (Bonferroni posttest)**		**Sample Size**	
**Time (h)**	**Control vs Heat shock**	**Control**	**Heat shock**
2	>0.05	6	6
24	>0.05	6	6
96	>0.05	6	6

Detailed statistics for Additional File 5. B16 tumors and clock gene expression.

Panel F: Relative circadian clock gene expression in the liver of DEX- or PBS-treated mice harboring tumors generated by s.c. injection of B16 cells.

1. **Cosine wave regression**

	p-value (F-test)		
**Gene**	**PBS**	**DEX**	**Sample size**
Bmal1	0.0439	0.002	9 (PBS), 10 (DEX)
Per1	0.1917	0.348	10
Per2	0.0793	0.174	9 (PBS), 10 (DEX)
Cry1	0.111	0.0101	10
Nr1d1	0.0504	0.064	9 (PBS), 10 (DEX)

2. **2-way ANOVA**

	p-value (2-way ANOVA)		
**Gene**	**Interaction**	**Group**	**Time**
Bmal1	0.174	0.704	<0.0001
Per1	0.0008	0.273	0.0027
Per2	0.998	0.274	<0.0001
Cry1	0.755	0.416	0.0005
Nr1d1	0.989	0.877	<0.0001

Detailed statistics for Additional File 8. Knockdown of *Bmal1* prevents the dexamethasone effects on B16 cell proliferation, cell cycle arrest and cell cycle phases.

Panel A, B: Cell cycle arrest of Scrambled shRNA-transfected B16 cells or *Bmal1* shRNA-transfected B16 cells.

1. **Scrambled shRNA: 2-way ANOVA**

**G0 phase Scrambled shRNA: p-value (2-way ANOVA)**			
**Interaction**	**Group**	**Time**	
0.4315	< 0.0001	0.0020	
**p-value (Bonferroni posttest)**		**Sample Size**	
**Time (h)**	**-DEX versus +DEX**	**-DEX**	**+DEX**
24	<0.05	5	5
48	<0.01	6	6
72	<0.001	6	6

**G1 phase Scrambled shRNA: p-value (2-way ANOVA)**			
**Interaction**	**Group**	**Time**	
0.7588	< 0.0001	0.0006	
**p-value (Bonferroni posttest)**		**Sample Size**	
**Time (h)**	**-DEX versus +DEX**	**-DEX**	**+DEX**
24	<0.001	5	5
48	<0.001	6	6
72	<0.001	6	6

**S/G2/M phase Scrambled shRNA: p-value (2-way ANOVA)**			
**Interaction**	**Group**	**Time**	
0.9423	< 0.0001	< 0.0001	
**p-value (Bonferroni posttest)**		**Sample Size**	
**Time (h)**	**-DEX versus +DEX**	**-DEX**	**+DEX**
24	<0.05	5	5
48	<0.05	6	6
72	<0.01	6	6

2. ***Bmal1* shRNA: 2-way ANOVA**

**G0 phase *Bmal1* shRNA: p-value (2-way ANOVA)**			
**Interaction**	**Group**	**Time**	
0.0986	0.1767	0.7180	
**p-value (Bonferroni posttest)**		**Sample Size**	
**Time (h)**	**-DEX versus +DEX**	**-DEX**	**+DEX**
24	>0.05	5	5
48	>0.05	6	6
72	>0.05	6	6

**G1 phase *Bmal1* shRNA: p-value (2-way ANOVA)**			
**Interaction**	**Group**	**Time**	
0.3606	0.9683	0.0050	
**p-value (Bonferroni posttest)**		**Sample Size**	
**Time (h)**	**-DEX versus +DEX**	**-DEX**	**+DEX**
24	>0.05	5	5
48	>0.05	6	6
72	>0.05	6	6

**S/G2/M phase *Bmal1* shRNA: p-value (2-way ANOVA)**			
**Interaction**	**Group**	**Time**	
0.3536	0.5209	0.6043	
**p-value (Bonferroni posttest)**		**Sample Size**	
**Time (h)**	**-DEX versus +DEX**	**-DEX**	**+DEX**
24	>0.05	5	5
48	>0.05	6	6
72	>0.05	6	6

Panel C-E: Cell cycle phases of Scrambled and *Bmal1* shRNA transfected B16 cells after DEX treatment.

1. **Cosine wave regression**

	**p-value (F-test)**		
**Phase**	**Scrambled shRNA**	***Bmal1* shRNA**	**Sample size**
G0/G1	0.001	0.657	24 (Scr.), 23 (*Bmal1*)
G2/M	0.291	0.342	24 (Scr.), 23 (*Bmal1*)
S	0.001	0.951	24 (Scr.), 23 (*Bmal1*)

2-way ANOVA

**G0/G1 phase: p-value (2-way ANOVA)**			
**Interaction**	**Group**	**Time**	
0.0003	0.0022	0.0063	
**p-value (Bonferroni posttest)**		**Sample Size**	
**Time (h)**	**Scrambled vs *Bmal1* shRNA**	**Scrambled shRNA**	***Bmal1* shRNA**
24	<0.001	6	5
30	> 0.05	6	6
36	> 0.05	6	6
42	> 0.05	6	6

**G2/M phase: p-value (2-way ANOVA)**			
**Interaction**	**Group**	**Time**	
0.0032	0.0019	0.4319	
**p-value (Bonferroni posttest)**		**Sample Size**	
**Time (h)**	**Scrambled vs *Bmal1* shRNA**	**Scrambled shRNA**	***Bmal1* shRNA**
24	> 0.05	6	5
30	<0.01	6	6
36	> 0.05	6	6
42	> 0.05	6	6

**S phase: p-value (2-way ANOVA)**			
**Interaction**	**Group**	**Time**	
0.0101	<0.0001	0.0015	
**p-value (Bonferroni posttest)**		**Sample Size**	
**Time (h)**	**Scrambled vs *Bmal1* shRNA**	**Scrambled shRNA**	***Bmal1* shRNA**
24	<0.001	6	5
30	<0.01	6	6
36	> 0.05	6	6
42	<0.001	6	6

Primers used for quantitative PCR

Gene	Forward primer (5'-3')	Reverse primer (5'-3')
Clock genes		
Bmal1	CCTAATTCTCAGGGCAGCAGAT	TCCAGTCTTGGCATCAATGAGT
Per1	TGGCTCAAGTGGCAATGAGTC	GGCTCGAGCTGACTGTTCACT
Per2	GCCAAGTTTGTGGAGTTCCTG	CTTGCACCTTGACCAGGTAGG
Cry1	GTCATTGCAGGAAAATGGGAAG	TAAAGAGGCGGAGAGACAAAGG
Nr1d1	AGCTCAACTCCCTGGCACTTAC	CTTCTCGGAATGCATGTTGTTC
Cell-cycle genes		
Wee1	GAAACAAGACCTGCCAAAAGAA	GCATCCATCTAACCTCTTCACAC
c-Myc	TGATGTGGTGTCTGTGGAGAAG	CGTAGTTGTGCTGGTGAGTGG
p21	GCAGACCAGCCTGACAGATTT	GAGAGGGCAGGCAGCGTAT
Cdk1	GGCAGTTCATGGATTCTTCACTC	GCCAGTTTGATTGTTCCTTTGTC
Cdk2	AAGGGCTGAGCTCTCCTTG	GGGAACCCTGACGAAAGC
Cyclin E	CCTCCAAAGTTGCACCAGTT	CCACTTAAGGGCCTTCATCA
Control gene		
Ef1a	TGCCCCAGGACACAGAGACTTCA	AATTCACCAACACCAGCAGCAA
