# Supplementary material for: Enhancing circadian clock function in cancer cells inhibits tumor growth
Source: BMC Biol. 2017 Feb 14;15:13. doi: 10.1186/s12915-017-0349-7 (PMC5310078; doi:10.1186/s12915-017-0349-7)
Supplement: Additional file 2: — Supplementary methods. (PDF 184 kb) [file 12915_2017_349_MOESM2_ESM.pdf]

Additional File 2: Methods

Cell culture and stable transfection. The B16 cells were cultured in RPMI with 10% (v/v) fetal bovine serum (FBS), 50 U/mL penicillin, 50 µg/mL streptomycin, 2 mM L-glutamine. The HCT-116 cells were cultured in McCoy's 5A Medium with 10% (v/v) FBS, 50 U/mL penicillin, 50 µg/mL streptomycin. Cells were treated with 50% horse serum in RPMI, 200 nM DEX, or 100 nM FSK for 2 h. In some experiments, there were additional DEX or FSK treatments at ~2 days intervals, as indicated. In other experiments, a heat shock was performed by incubating cells at 43°C for 30 min. For gene expression profiles, cells were harvested at the indicated times using 0.25% Trypsin, and frozen in PBS until subsequent processing. All culture reagents were from Life Technologies.

B16 cells were transfected with pGL4.16 vector (Promega, Madison, WI, USA) expressing luciferase (luc2) under the control of *Bmal1* or *Per2* promoter, using Lipofectamine 2000 (Life Technologies, Carlsbad, CA, USA) as per manufacturer's protocol. For stably-transfected cell selection, 24 h after transfection, cells were replated in medium with hygromycin (4 µg/mL). Colonies were transferred to 6-well plates, grown for 24 h without antibiotic and then in medium with hygromycin. Resistant clones were tested for optimal bioluminescence and the best clones were used for further experiments.

Bioluminescence recordings. For whole-culture imaging, 1.5×10^6 B16 cells were plated in 3.5-cm dishes containing growth medium supplemented with 0.1 mM luciferin potassium salt (Promega). Explanted tissue slices (200 µm) of tumors from B16 cells with luciferase reporters were cut on a vibratome and placed on Millipore membranes (Millipore, Darmstadt, DE) and then plated in 3.5-cm dishes containing growth medium supplemented with 1 mM luciferin

potassium salt (Promega). Due to the initial burst of bioluminescence after medium change, the first 12 h of data were excluded from analysis. The overall brightness was calculated by averaging values from 12 h after start of recording to the end of recording. A linear baseline was subtracted from raw data (polynomial order = 1). Circadian rhythmicity was assessed by spectral analysis using the FFT algorithm in LumiCycle Analysis (Actimetrics, Wilmette, IL, USA). Cells were considered to show significant circadian rhythmicity when there was at least one spectral peak in the circadian range (20–28 h period).

For single-cell imaging, B16 cells or explant tissues were cultured as described above and placed on a custom-built microscope inside an incubator set at 35°C. Light from the sample was collected above the sample stage by a Union NPLM 20X objective (NA 0.35; WD=15 mm, tube length of 30 mm, Mag 2.86x) mounted 30 mm from a charge-coupled device camera sensor (Ikon-M 934BV Series, Andor Technology, Belfast, UK). The signal-to-noise ratio was increased by 2 × 2 binning. Images were collected at intervals of 30 min, with 29 min exposure duration. Images were acquired using Andor Solis software (Andor Technology) and analyzed with MetaMorph (Molecular Devices, Sunnyvale, CA, USA) and cosmic ray artifacts were removed by using ImageJ (National Institute of Health, Bethesda, MD, USA). Data were smoothed by a running minimum algorithm, and bioluminescence intensity measured within a region of interest with a constant size, but defined manually for each moving cell. Luminescence intensity values were corrected by subtracting the intensity of a background region devoid of cells.

Proliferation, apoptosis and cell cycle assays. To count live and dead cells *in vitro*, cells attached to the dish and cells floating in the medium were harvested at the indicated times, washed with PBS, mixed with Trypan blue 0.4% (2:1), and counted with a hemocytometer.

To assess apoptosis by Annexin V staining (1), B16 and HCT-116 cells were treated with DEX or heat shock as above, and detached at the indicated times, or cells from s.c. tumors in NSG and C57BL/6J (collected as described above), were washed with PBS and incubated 15 min at room temperature (RT) in 10 µL binding buffer (100 mM HEPES, 140 mM NaCl,. 25 mM CaCl2) and 0.1 µL FITC Annexin V (#640906, BioLegend, San Diego, CA, USA). Samples ($2.5x10^4$ cells) were analyzed by flow cytometry, using a FACSCalibur (BD Biosciences, Franklin Lakes, NJ, USA) and FlowJo software (FlowJo, LLC, Ashland, OR, USA) (same thing for all other flow cytometry experiments, below).

To determine cell cycle phases, BrdU was added to the medium (0.6 µg/mL) for 2 h before harvesting at the indicated times. Cells were washed in PBS, fixed with 70% ethanol for 30 min at -20°C, washed in PBS containing 1% Triton X-100 (PBST), and then incubated in 1 N HCl solution on ice for 10 min. Cells were incubated in 2 N HCl solution at RT for 10 min and then 37°C for 20 min. PBS was added to the cells for 10 min, which were then washed with PBST and blocked in PBST with 1% BSA and 1 M glycine for 1 h. Cells were incubated overnight at 4°C in 100 µL PBST with 1% BSA and mouse anti-BrdU antibody (RRID:AB_305426, #ab6326, Abcam, Cambridge, UK) (1:500). Cells were then washed with PBST and incubated for 1 h at RT in 100 µL PBST with 1% BSA and Alexa-fluor 488 goat anti-mouse IgG (#ab150157, Abcam) (1:400). Finally, the BrdU-stained cells were washed in PBS, resuspended in 100 µL binding buffer, incubated with 0.05 µg/mL 7AAD viability staining solution

(#420404, Biolegend, San Diego, CA, USA) for 20 min at RT. Samples ($2.5x10^4$ cells) were analyzed by flow cytometry as above.

Tumor collection and homogenization. Tumors were collected throughout the circadian day (2nd day in constant darkness) on the 7th day after the first DEX treatment for C57BL/6J mice and when tumors have reached reached >50% less tumor volume than PBS-injected tumors for NSG mice. Cells were homogenized using a cell strainer (Thermo Fisher, Carlsbad, CA, USA). One half of the cells were resuspended in PBS (CD4, CD8, F4/80, CD11c stainings), while the other half was resuspended in PBS containing 1% BSA and 2.5% collagenase II (Invitrogen, Carlsbad, CA, USA) and digested at 37°C for 13 min (for CD11b, CD19, LygG, Ly6C, F4/80 stainings). They were then washed with RPMI 10% FBS and finally resuspended in RPMI. Cells were then incubated for 5 min at RT with 0.83% NH_4Cl to lyse the red blood cells and washed with RMPI. Then all cell suspensions were fixed in 4% paraformaldehyde for 10 min and washed in PBS. Permeabilization was done with 90% methanol at -20°C. Cells were stored at -80°C until further staining.

Tumor immune cell infiltration in vivo. One million cells from s.c. tumors (with and without collagenase treatment, collected as described above) were washed with PBS/1% BSA and blocked with Fc Block (RRID:AB_1574975, #101320, Biolegend, San Diego, USA) on ice for 10 min, washed again with PBS/1%BSA, and then incubated on ice for 20 min with: PE anti-CD4 (RRID:AB_312693, #100408) and PerCP anti-CD8 (RRID:AB_893423, #100732) to measure frequency of T cells; Alexa 647 anti-CD19 (RRID:AB_389329, #115522) for B cells; APC anti-CD11c (RRID:AB_313779, #117310) for dendritic cell; APC anti-F4/80

(RRID:AB_893481, #123116) and biotin anti-CD11b (RRID:AB_312787, #101204) for macrophages; biotin anti-CD11b, APC anti-F4/80 and FITC anti-Ly6G (RRID:AB_1236488, #127605) for neutrophils; biotin anti-CD11b, APC anti-F4/80 and Alexa 488 anti-Ly6C (RRID:AB_10640820, #128021) for monocytes (all antibodies from Biolegend). For CD11b staining, cells were washed with PBS/1% BSA and incubated with PerCP-conjugated streptavidin (#405213, Biolegend) for 20 min on ice. Finally cells were washed with PBS or PBS/1% BSA and stored at 4°C until analysis. The samples were analyzed by flow cytometry as above.

Protein expression in vivo. For protein expression homogenized tumor cells (collected as described above) were fixed in 4% paraformaldehyde, permeabilized with 90% methanol. One million cells were incubated with antibodies for WEE1, c-MYC, p57 (all from rabbit, RRID:AB_2288509, #4936, RRID:AB_1903938, #5605, RRID:AB_2291591, #2557, Cell Signaling, Cambridge, UK), CYCLIN E or CDK2 (both from rabbit, RRID:AB_627362, #sc-248, RRID:AB_631215, #sc-163, Santa Cruz, Dallas, USA) in PBS, or antibodies for CDK1 (from mouse, RRID:AB_2074906, #ab18, Abcam, Cambridge, UK), p21 (from mouse, #NBP2-29463, clone: WA-1 (HJJ21)) or BMAL1 (from rabbit, RRID:AB_10000794, #NB100-2288, both Novus Biologicals, Littleton, USA) in PBS/1% BSA, for 1 h at RT, then washed, and blocked with 10% normal rat or goat serum depending on the secondary antibody (30 min, RT). The secondary antibody (Alexa Fluor 647 goat anti-mouse [#405321, Biolegend] for CDK1; preadsorbed APC rat anti-mouse IgG1 [RRID:AB_2573205, #17-4015, eBioscience, San Diego, CA, USA] for p21; Alexa Fluor 488 donkey anti-rabbit [RRID:AB_2563203, #406416, Biolegend] for all others) was added to the cells for 20 min at RT. Controls for each sample were

stained with secondary antibody alone. Cells were washed with PBS or PBS/1% BSA and stored at 4°C until analysis. The samples were analyzed by flow cytometry as above.

Cell cycle assays in vivo. Cell cycle phases were determined using BrdU and 7AAD staining. Cells from tumors (without collagenase treatment, collected as described above) were washed in PBST/1% BSA and incubated overnight at 4°C in 100 µL PBST/1% BSA with mouse anti-BrdU antibody (RRID:AB_305426, #ab6326, Abcam, Cambridge, UK) (1:500). Cells were then washed with PBST and incubated for 1 h at RT in 100 µL PBST/1% BSA with Alexa-fluor 488 goat anti-mouse IgG (#ab150157, Abcam) (1:400). Finally, the BrdU-stained cells were washed in PBS, resuspended in 100 µL binding buffer, incubated with 0.05 µg/mL 7AAD viability staining solution (#420404, BioLegend) for 20 min at RT. The samples were analyzed by flow cytometry as above.

Mitotic index was assessed on cells from tumors (without collagenase treatment, collected as described above). The cells were incubated with anti-pHH3 antibody (RRID:AB_331534, #9701, Cell signaling, Cambridge, UK, 1:50) in PBS/1% BSA for 30 min at RT, washed with PBS/1% BSA and incubated with Alexa Fluor 488 donkey anti-rabbit antibody (RRID:AB_2563203, #406416, Biolegend, 1:5000) for 30 min at RT. Controls for each sample were stained with secondary antibody alone. The samples were analyzed by flow cytometry as above.

Cell cycle arrest and proliferation were detected in cells from tumors (without collagenase treatment, collected as described above) in NSG mice using the Ki67 Set according to manufacturer's protocol (RRID:AB_2266296, #556027, BD Biosciences). Cells were permeabilized with Permbuffer for 10 min at RT. After washing, cells were incubated for 20 min at RT in darkness with Ki67 (10 µL/10^6 cells), washed and resuspended in 100 µL binding

buffer, incubated with 0.05 µg/mL 7AAD viability staining solution (BioLegend) for 20 min at RT. The samples were resuspended in 200 µL FACSWash. Controls for each sample were stained with the secondary antibody alone. Samples were analyzed by flow cytometry as above.

Quantitative PCR. Total RNA from tumors, livers, lungs, B16 cells and HCT-116 cells was isolated using Trizol (Life Technologies, Carlsbad, CA, USA) according to the manufacturer's protocol. RNA concentration and purity were determined with an ND-1000 spectrophotometer (NanoDrop, Thermo Fisher, Carlsbad, CA, USA). Complementary DNA was synthesized using the High Capacity cDNA Reverse Transcription Kit (#4368814, Applied Biosystems, Foster City, CA, USA) according to the manufacturer's protocol. Quantitative PCR was performed in a 7500 Real Time PCR System (Life Technologies) using GoTaq qPCR Mastermix (#A6002, Promega) according to the manufacturer´s protocol. The list of primer sequences of clock genes and cell cycle genes used is provided in Additional File 1. *Elongation factor 1-alpha* was used as reference gene and relative quantification of expression levels were performed as described (2).

Immunohistochemistry. Eight µm O.C.T. (Sakura-Finetek, Alphen aan den Rijn, NE)-embedded cryosections were processed for immunohistochemistry for BMAL1 and DAPI staining as described (3). The anti-BMAL1 antibody (RRID:AB_10000794, #NB100-2288, Novus Biologicals, Littleton, CO, USA) and Alexa-fluor 488 goat anti-rabbit secondary antibody (RRID:AB_2534114, #A11070, Life Technologies) were diluted 1:1000 and 1:400, respectively. Pictures were taken with an Observer.Z1 fluorescent microscope (Zeiss, Jena, DE) and analyzed with the ImageJ software (National Institutes of Health). Three sections from different areas within the tumors were used and for each tumor the background from adjacent areas on the same

slide was subtracted. BMAL1-positive cells were counted and calculated relative to the numbers of DAPI-stained cells.

Population doubling time analysis

Population doubling time (PDT) in hours was calculated during the individual exponential growth phase of each cell culture based on the equation: $\left[PDT = \frac{t2 - t1}{3.32} * \left(\log n(t2) - \log n(t1)\right)\right]$ where *n(t2)* represents the cell yield at the last time point *(t2)* and *n(t1)* indicates the cell number used to begin that subculture and the starting time point *(t1)*.

Tumor volume doubling time analysis. Tumor volume doubling time (VDT) in days was calculated during the individual exponential growth phase of each tumor (VDT = ln $^{(2) / k}$) based on the equation: a cosine wave equation $\left[Y = Y_0 * \left(\exp\left(k * X\right)\right)\right]$ where Y_0 represents the starting volume and k indicates the rate constant.

Additional References

1. Vermes I, Haanen C, Steffens-Nakken H, and Reutelingsperger C. A novel assay for apoptosis. Flow cytometric detection of phosphatidylserine expression on early apoptotic cells using fluorescein labelled Annexin V. *J Immunol Methods* **1995**;184:39-51.
2. Ramakers C, Ruijter JM, Deprez RH, and Moorman AF. Assumption-free analysis of quantitative real-time polymerase chain reaction (PCR) data. *Neurosci Lett* **2003**;339:62-66.
3. Husse J, Zhou X, Shostak A, Oster H, and Eichele G. Synaptotagmin10-Cre, a driver to disrupt clock genes in the SCN. *J Biol Rhythms* **2011**;26:379-389.
