# Supplementary figures and images for: Enhancing circadian clock function in cancer cells inhibits tumor growth
Source: BMC Biol. 2017 Feb 14;15:13. doi: 10.1186/s12915-017-0349-7 (PMC5310078; doi:10.1186/s12915-017-0349-7)

# Additional File 4

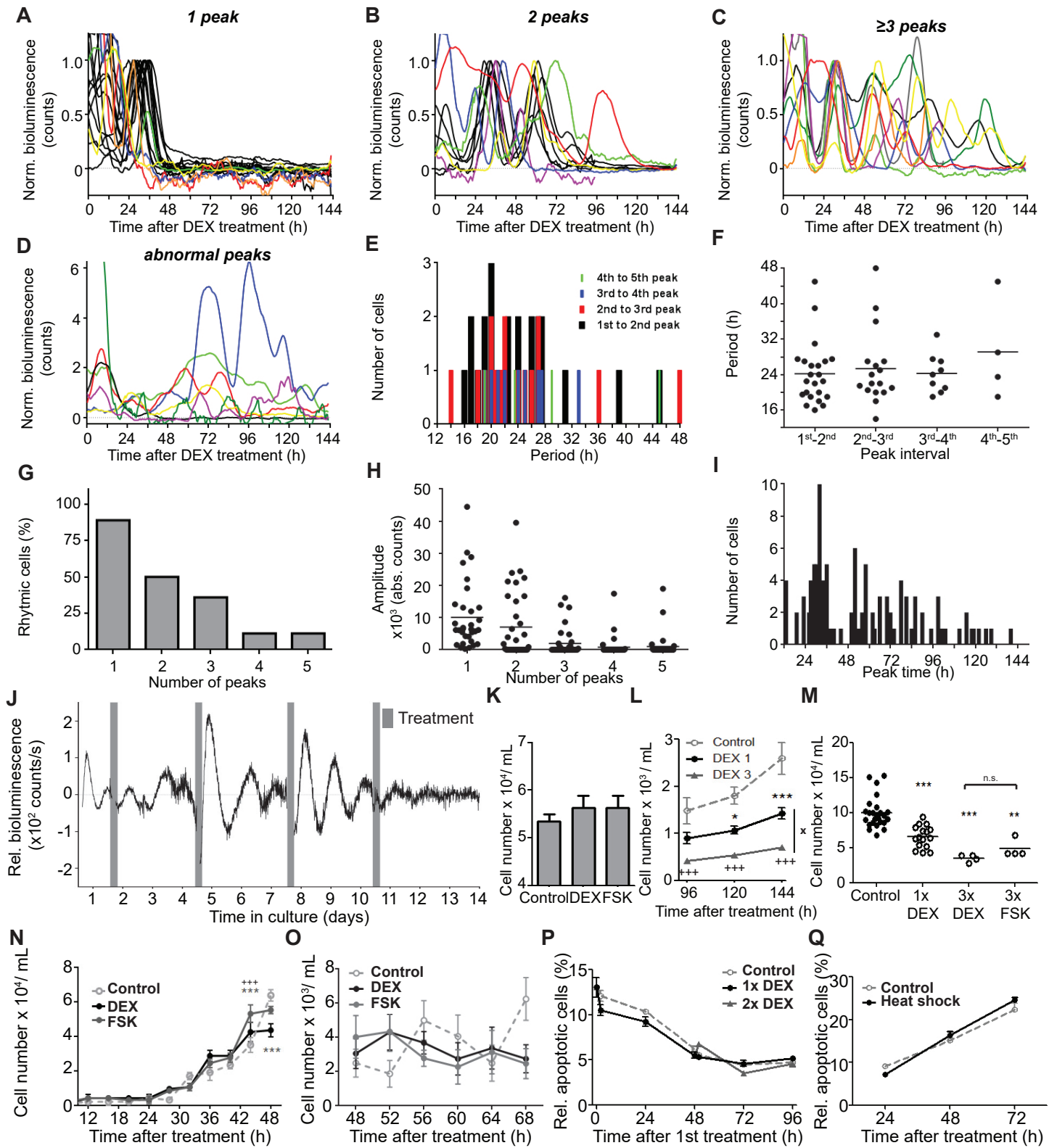

Supplement: Additional file 4: — Single-cell analysis of Per2-Luc B16 cells and level of apoptosis after treatment. Bioluminescence of single Per2-Luc B16 cells after DEX treatment, grouped depending on their number of circadian peaks: (A) one peak (n = 19), (B) two peaks (n = 10), (C) three or more peaks (n = 9) and (D) abnormal and non-circadian peaks (n = 6). (E) Period distribution of single Per2-Luc B16 cells after DEX treatment. Analysis of circadian period (F), rhythmicity (G), amplitude (H) and peak time (I) of single Per2-Luc B16 cells. (J) Bioluminescence of Per2-Luc B16 cells undergoing repeated treatments (grey bars) after plating: medium change (day 1), DEX (day 4, 7) and FSK (day 10). (K) B16 cells were counted before, 2 h after treatment with DEX or FSK (untreated: n = 24, DEX: n = 6, FSK: n = 6, Kruskal-Wallis test, p > 0.05), (L) after a single or three DEX treatments and compared to untreated controls (n = 6 wells/time point, two-way ANOVA p < 0.001, posthoc test, *p < 0.05, ***p < 0.001 DEX1 compared to controls, +++ p < 0.001 DEX3 compared to controls, x p < 0.05 DEX1 compared to DEX3). (M) B16 cells were counted after a single or three DEX or FSK treatments and compared to untreated cells (control n = 25, DEX1: n = 16, DEX3: n = 4, FSK3: n = 4, Kruskal-Wallis test, Dunn’s multiple comparison test, **p < 0.01, ***p < 0.001). (N, O) Total dead cells in the medium 12–48 h (N) and 48–68 h (O) after treatment with either DEX or FSK and controls (n = 6–8 wells/time point). (P) Apoptotic cells stained for Annexin V 0–96 h after control or DEX treatment at 0 h (DEX1) or at 0 and 48 h (DEX2) (n = 4–12 wells/time point). (Q) Apoptotic cells stained for Annexin V 0–72 h after heat shock or control treatment (n = 6 wells/time point). Two-way ANOVA, posthoc test, ***p < 0.001 DEX compared to controls, +++ p < 0.001 FSK compared to controls. For details of statistics, see Additional file 1. (PDF 1581 kb) [file 12915_2017_349_MOESM4_ESM.pdf]

Additional File 5

A

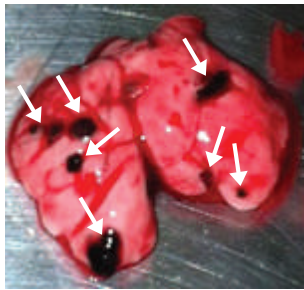

B

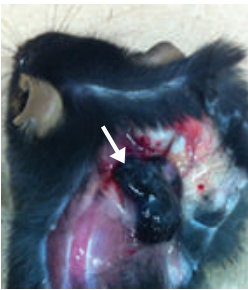

E

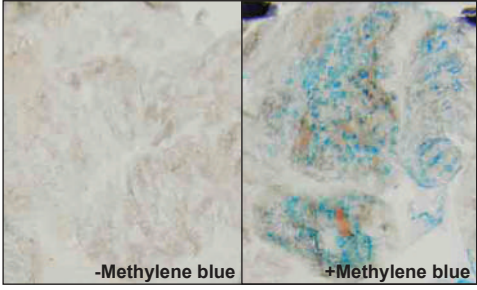

C

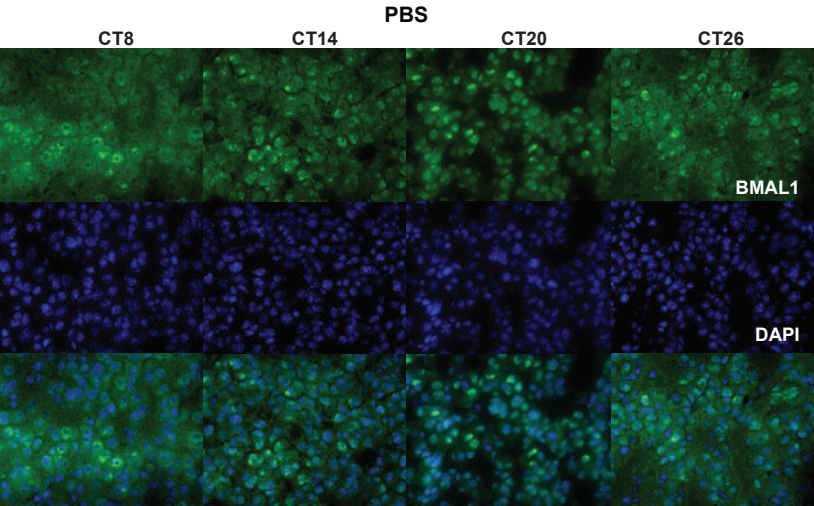

D

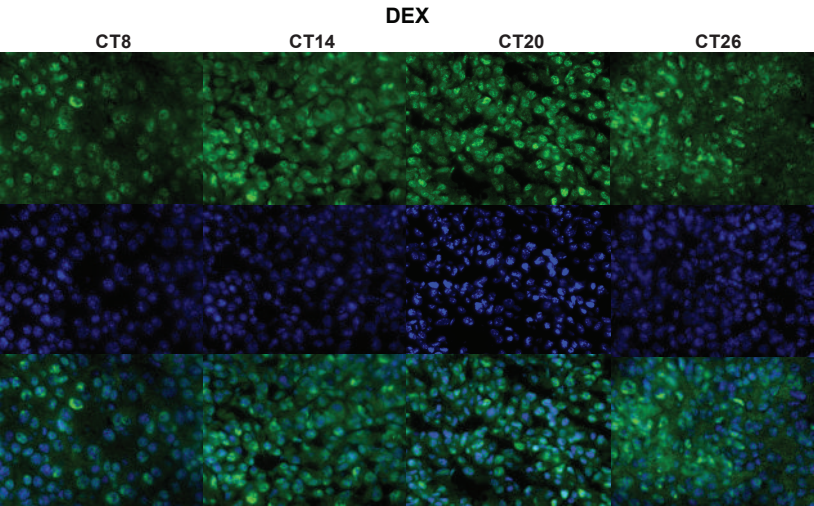

F

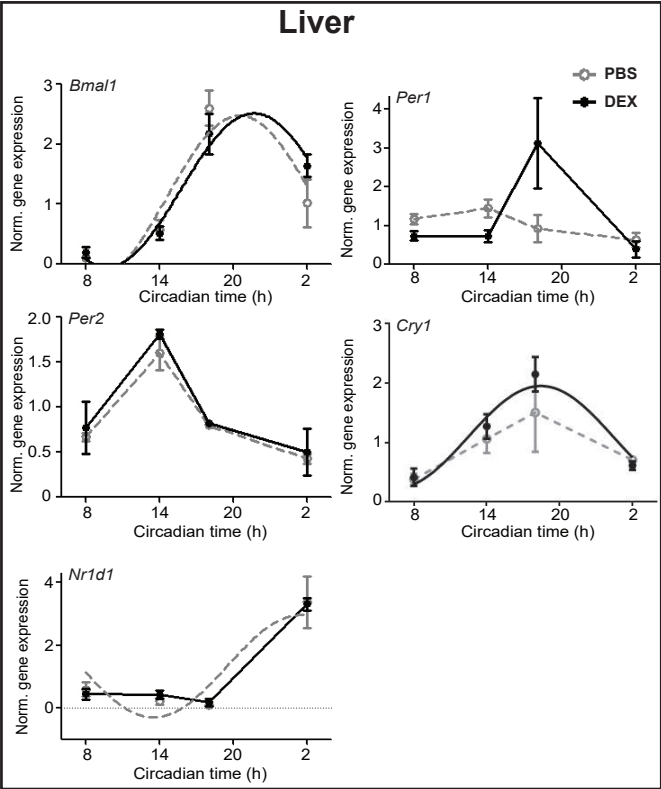

Supplement: Additional file 5: — B16 tumors and clock gene expression. (A) Lung tumors (black, indicated by arrows) formed after injection of 1.5 × 10^6^ B16 cells in the tail vein, within the surrounding lung tissue (pink). (B) B16 tumors (black, indicated by arrow) formed after subcutaneous injection of 1.5 × 10^6^ B16 cells in the neck. (C, D) Representative immunohistochemistry images for BMAL1 (green) and DAPI (blue) from PBS-treated (C) and DEX-treated (D) tumors collected at CT2, 8, 14 and 20. See full data in Fig. 3j. (E) Methylene blue (200 μL of 1% in saline) was injected intra-tumorally. Six hours later, tumors were harvested, sectioned and visualized by light microscopy. (F) Relative circadian clock gene expression in the liver of DEX- or PBS-treated mice harboring tumors generated by s.c. injection of B16 cells (n = 9–10, 2–3 mice/time point; cosine-wave regression, F-test: -DEX: Bmal1 and Nr1d1: p < 0.05, +DEX: Bmal1 and Cry1: p ≤ 0.01, Per1: p = 0.348, Per2: p = 0.174, Nr1d1: p = 0.0646; two-way ANOVA: group: all genes: p > 0.05, time: Bmal1, Per2, Cry1 and Nr1d1: p < 0.001; Per1: p < 0.01). Significant rhythms are illustrated with fitted cosine curves, otherwise data are connected by straight lines between data points, indicating no significant circadian rhythms. Data are represented as mean ± SEM. For details of statistics, see Additional file 1. (PDF 8 MB) [file 12915_2017_349_MOESM5_ESM.pdf]

Additional File 7

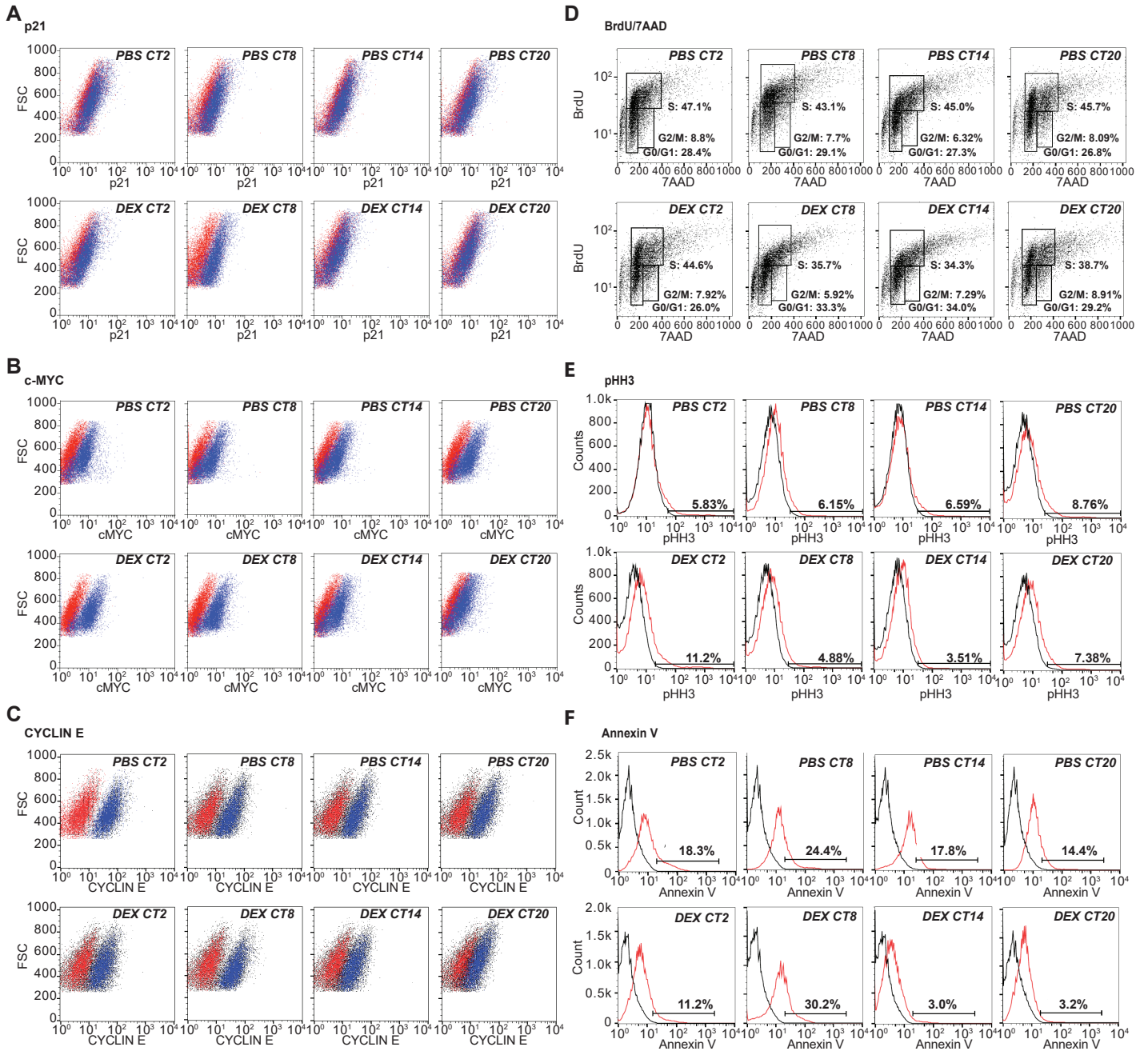

Supplement: Additional file 7: — DEX induces rhythmic cell cycle protein expression and cell cycle phases, mitosis and apoptosis. Representative flow cytometry dot plots for p21 (A), c-MYC (B) and CYCLIN E (C) protein expression at the indicated CTs in s.c. tumors after repeated intra-tumoral DEX or PBS injection every 2 days for 8–11 days. Unstained control (red) and stained (blue) cells of the indicated antibody. (D) Representative flow cytometry dot plots for incorporated BrdU and staining with 7AAD at the indicated CTs in s.c. tumors after repeated intra-tumoral DEX or PBS injection every 2 days for 8–11 days. The illustrated gates indicate the analyzed cell cycle phases. Representative histogram for pHH3 (E) and histogram for Annexin V staining (F) at the indicated CTs in s.c. tumors after repeated intra-tumoral DEX or PBS injection every 2 days for 8–11 days. Positive (red) cells compared to unstained control cells (black). (PDF 2 MB) [file 12915_2017_349_MOESM7_ESM.pdf]

Additional File 8

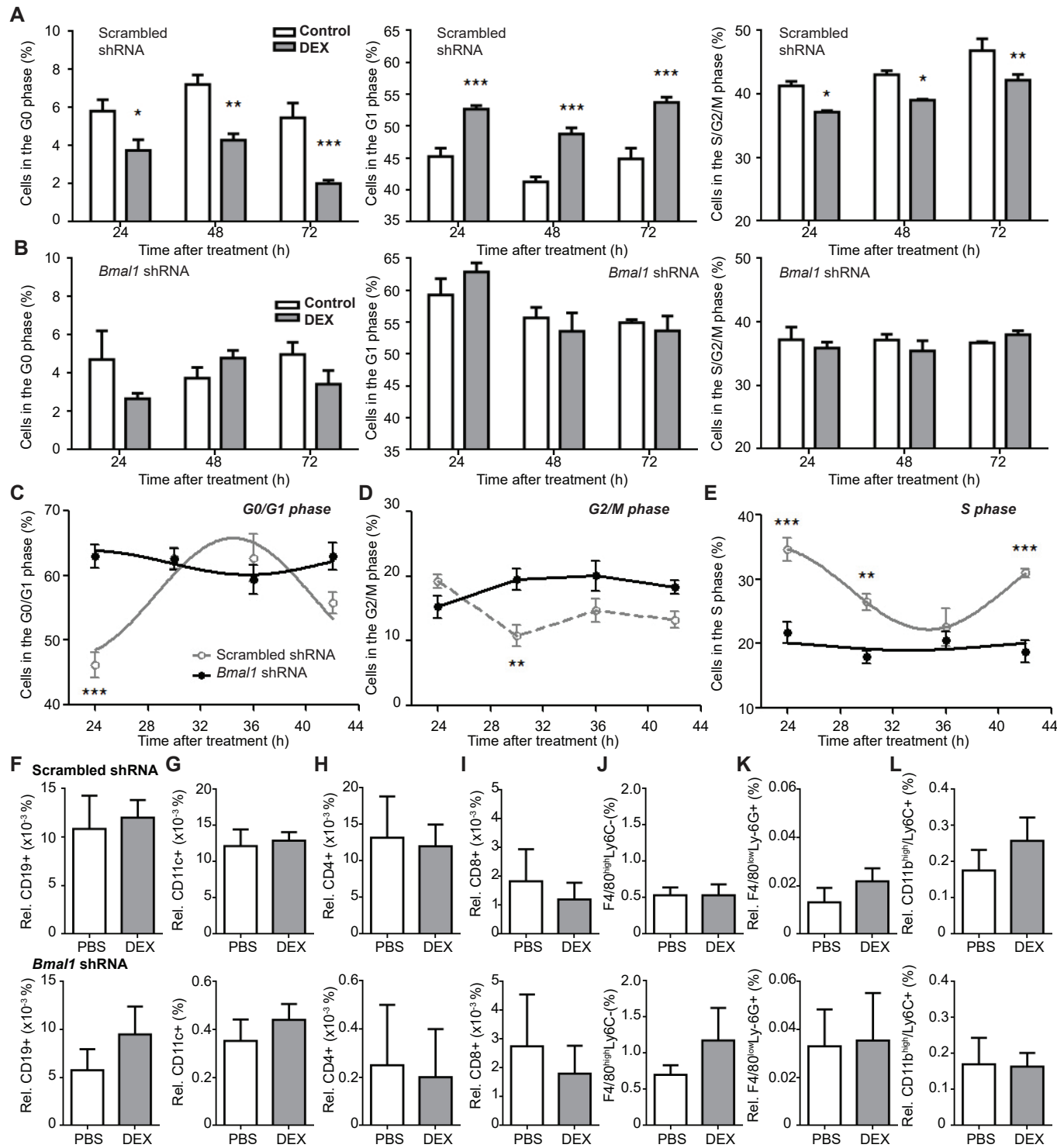

Supplement: Additional file 8: — Knockdown of Bmal1 prevents the DEX effects on B16 cell proliferation, cell cycle arrest and cell cycle phases. (A, B) Cell cycle arrest of Scrambled shRNA-transfected B16 cells (A) or Bmal1 shRNA-transfected B16 cells (B) indicated by cells in the G0 phase (left panels) and cell proliferation indicated by cells in the G1 phase (middle panels) or S/M/G2 phases (right panels) with control or DEX treatment (n = 5–6, wells/time point/group; two-way ANOVA, posthoc test, group effect: *p < 0.0.5, **p < 0.01, ***p < 0.001). (C–E) Cell cycle phases of Scrambled shRNA- and Bmal1 shRNA-transfected B16 cells after DEX treatment. Significant rhythms are illustrated with fitted cosine curves, otherwise data are connected by straight lines between data points, indicating no significant circadian rhythms (n = 10–15, 3–4 wells/time point; cosine-wave regression, F-test: p < 0.05; two-way ANOVA, posthoc test, group effect: **p < 0.01, ***p < 0.001). Frequencies of (F) B cells (CD19+), (G) dendritic cells (CD11c+), (H) CD4 T cells (CD4+), (I) CD8 T cells (CD8+), (J) macrophages (F4/80highLy6C-, gated on CD11b+), (K) neutrophils (F4/80 lowLy6Ghigh, gated on CD11b+) and (L) monocytes (CD11bhighLy6C+, gated on F4/80+) relative to alive cells after intra-tumoral PBS or DEX injection in B16 s.c. tumors of C57BL/6 J mice (Scrambled shRNA tumors and Bmal1 shRNA tumors: no significant difference between DEX and PBS throughout the experiment, n = 5–6 mice/group, Mann-Whitney rank sum test: p > 0.05). Data are represented as mean ± SEM. For details of statistics, see Additional file 1. (PDF 844 kb) [file 12915_2017_349_MOESM8_ESM.pdf]

Additional File 9

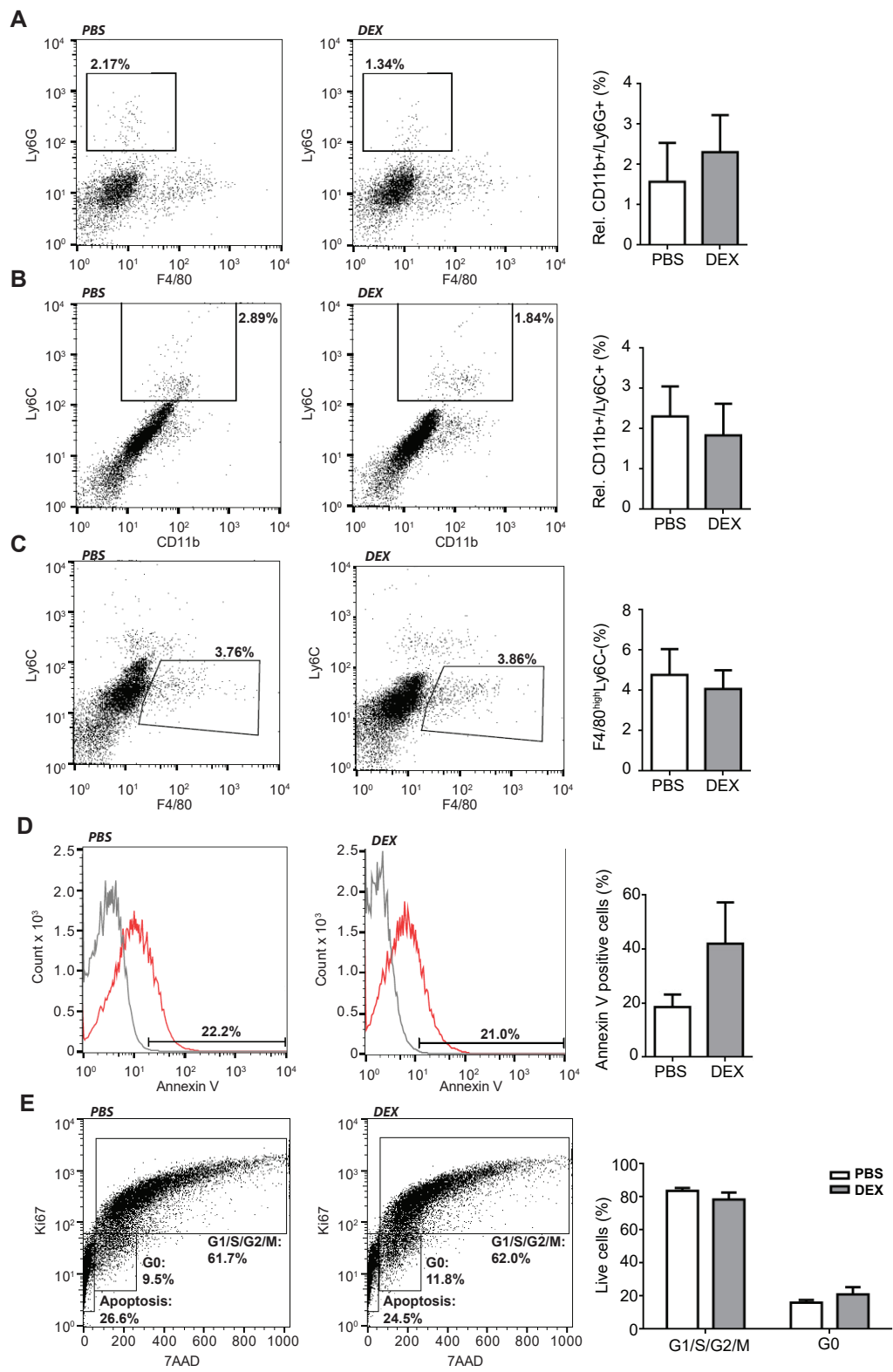

Supplement: Additional file 9: — Immune cell infiltration, apoptosis and cell cycle arrest in NSG mice. Representative flow cytometry dot plots and quantification for neutrophils (F4/80 lowLy-6Ghigh, gated on CD11b+) (A), monocytes (CD11bhighLy-6Chigh, gated on F4/80+) (B), macrophages (F4/80highLy-6C- gated on CD11b+) (C), histogram and quantification for apoptosis (Annexin V) stained (red) and unstained (grey) cells (D) and dot plots and quantification of proliferation (Ki67) (E) at the indicated CTs in s.c. tumors after repeated intra-tumoral PBS or DEX injection every 2 days for 8–11 days in B16 s.c. tumors of NSG mice collected at CT14 (n = 4 mice/group, Mann-Whitney rank sum test: p > 0.05). (PDF 812 kb) [file 12915_2017_349_MOESM9_ESM.pdf]
